# The nitrovinyl moiety determines the cyto- and genotoxic profiles of β-nitrostyrene derivatives: evidence from in silico and in vitro evaluation

**DOI:** 10.1007/s00204-026-04349-4

**Published:** 2026-03-31

**Authors:** Jan Sündermann, Stella Marie Reamon-Buettner, Sabrina Wilde, Annette Bitsch, Christina Ziemann

**Affiliations:** 1 Fraunhofer Institute for Toxicology and Experimental Medicine ITEM, Nikolai-Fuchs-Straße 1, 30625 Hannover, Germany; 2 Lyomark Pharma GmbH, Keltenring 17, 82041 Oberhaching, Germany

**Keywords:** Genotoxicity, β-nitrostyrene, Structural similarity, QSAR, Clastogenicity, Cytotoxicity, Comet assay, γH2A.X, pH3 (S10)

## Abstract

β-Nitrostyrene, an important intermediate in chemical industry, shows relevant anti-proliferative and pro-apoptotic potential and has thus been suggested for potential use in tumor chemotherapy. If not derivatized, β-nitrostyrene is however, highly reactive and possess (geno)toxic potential. To decipher structural elements relevant for biological activity, crucial for both, chemical risk assessment and potential drug development, a panel of nine structural derivatives of β-nitrostyrene, was put together. It included structures with and without nitrovinyl moiety and was based on fingerprint similarity, QSAR-based mutagenicity alerts, and literature reports. It comprised simple structures like styrene and more complex structures such as 2-methoxy-5-[(E)-2-nitroethenyl]phenol. In vitro experiments in human WS1 and mouse L5178/TK^±^ cells demonstrated that the nitrovinyl moiety of β-nitrostyrene seems vital for its cytotoxic (membrane damage, metabolic competence) and genotoxic (clastogenicity, mutagenicity) properties. The extent of adversity varied with modification of structural elements. For example 1-nitro-2-[(E)-2-nitroethenyl]benzene led to 2.3-fold higher mean tail intensities in the alkaline comet assay than β-nitrostyrene. Notably, all derivatives with a nitrovinyl moiety, except 5-[(E)-2-nitroethenyl]-1,3-benzodioxole, induced DNA double-strand breaks, as judged by γH2A.X induction. In conclusion, our study identified structural elements responsible for the cyto- and genotoxic potential of β-nitrostyrene, demonstrating that minor structural changes can significantly alter its adverse biological activity.

**Supplementary Information:**

The online version contains supplementary material available at 10.1007/s00204-026-04349-4.

## Highlights

- Grouping approach to elucidate substructural elements responsible for cyto- and genotoxicity of β-nitrostyrene and structurally related compounds.
- Only compounds possessing a nitrovinyl moiety showed cytotoxic effects in L5178Y/TK^±^ and WS1 cells.
- Genotoxicity testing revealed that the nitrovinyl moiety is responsible for DNA double strand break induction, with one compound likely causing DNA single strand breaks only.
- Overall, the nitrovinyl moiety was key in induction of (geno)toxicity by β-nitrostyrene which could be increased in potency by modification of the phenyl-moiety of the lead-structure.

## Introduction

The industrial chemical β-nitrostyrene, which is, amongst others, used as a chain-length stopper in the free radical-mediated polymerization of styrene (Encinas et al. 2001) has gathered further attention, as it previously showed relevant antibiotic (Wang et al. 2024), anti-fungal (Brain et al. 1946; Ramzan et al. 2022) and in vivo anti-neoplastic properties in a Krebs II ascites tumor model (Doré and Viel 1975). In vitro and in vivo anti-neoplastic properties of various β-nitrostyrene derivatives were assessed in more recent publications (Tsai et al. 2017a; Wang et al. 2017a; Panina et al. 2022; Byrne et al. 2023; McKeown et al. 2024). Despite indications for a therapeutic potential of β-nitrostyrene derivatives, the structural elements responsible for its known (geno)toxic effects, remains only partly understood.

In this study, we employed a grouping approach to identify the biologically relevant structural elements of β-nitrostyrene. Typically, this approach serves a different purpose; it is widely used to fill data gaps in regulatory context i.e., as outlined by the European Chemicals Agency (ECHA) and the European Food Safety Authority (EFSA). Respective guidance for this approach is provided under the Registration, Evaluation, Authorisation and Restriction of Chemicals (REACh) regulation (The European Parliament and the Council of the European Union 2006; European Chemicals Agency ECHA 2017).

Here we applied similar principles and assumptions. Specifically, the formation of compound groups or chemical categories was justified by (bio)chemical properties that are either homogeneous or follow a pattern. The homogeneity of a compound group is assumed to stem from structural similarity, which can be attributed to "a common functional group" (Ball et al. 2016). However, it is important to note that compound grouping should not rely exclusively on structural similarity (Raies and Bajic 2016).

By testing a comprehensive compound panel, we were able to assess the impact of structural alterations of the lead compound on the chosen endpoints. Given the known (geno)toxic properties of β-nitrostyrene (Doré and Viel 1975), we selected endpoints that allow to draw conclusions regarding the mode of action of the test compounds. These endpoints included the phosphorylation of serin 139 on histone H2A.X (γH2A.X) or serin 10 on histone H3 (pH3 [S10]), detecting DNA double stand breaks or mitotic arrest, respectively (Khoury et al. 2016). This differentiation aids in distinguishing between the induction of structural chromosome changes/clastogenicity (van Maanen et al. 1988) or numerical chromosome changes/aneugenicity (Jordan 2002). The insights gained might contribute to a better understanding of the risk profile of β-nitrostyrene and β-nitrostyrene derivatives, and to the potential development of new cancer treatments.

## Methods

### Test compound selection and grouping approach

Biologically relevant (sub)structural elements were elucidated from a comprehensive panel of nine compounds, established through a stepwise selection process that included literature and database searches, chemical descriptors, fingerprint similarity and QSAR alerts assessment.

#### Literature search for candidates

The majority of the analog compounds were selected based on literature information, although this was done by a non-systematic literature review. Additionally, a similarity-driven database screening (HPVC List—United States High Production Volume Challenge Program List^1^) identified certain derivatives of interesting β-nitrostyrene. In total, 29 derivatives were gathered (Tab. S1).

#### Chemical descriptor assessment

The entire dataset (29 compounds) was characterized using a Konstanz Information Miner workflow (KNIME, Version 4.4.2) with the Chemistry Development Kit (CDK) and RDKit nodes, as depicted in Fig. S1. Both packages were used to generate fingerprint-based Tanimoto similarity values (CDK: PubChem, MACCS, Circular, Estate, Standard, Extended; RDKit: Pattern, Layered, MACCS, Avalon, Morgan, FeatMorgan, AtomPair, RDKit, Torsion) (Tab. S2). Molecular descriptors, such as partition coefficient (LogP), molecular weight, total polar surface area (TPSA), hydrogen-bond donors, hydrogen-bond acceptors and the number of heavy atoms (see Tab. S3 for overview), were calculated using the RDKit in KNIME.

#### Genotoxicity prediction

In parallel, mutagenicity alerts were investigated using various quantitative structure–activity relationship (QSAR) models, such as GT1_BMUT (statistical-based model, version 1.7.0.3.13514.500), PHARM_BMUT (statistical-based model version 1.7.0.5.17696.350) and GT_EXPERT (expert rule-based model, version 1.7.0.3.13514.500) within the CASE Ultra software (version 1.7.0.5).

#### Grouping and final test compound selection

The selection of the final nine test-compounds from the 29 identified derivatives was based on specific structural features and properties with the aim of assessing the structure activity relationship of β-nitrostyrene. Each test-compound was assigned an identification number, as listed in Table 1, according to the PubChem fingerprint Tanimoto similarity value generated by comparison to the lead-structure, which was “no. 1” in the following. These similarity values ranged from 0.88 to 0.58 for the test compounds and from 0.88 to 0.432 for the 29 derivatives.

**Table 1:** Selected β-nitrostyrene derivates for subsequent cyto- and genotoxicity testing with compound number, Tanimoto coefficient computed using the PubChem fingerprint (Tc), International Union of Pure and Applied Chemistry (IUPAC) name, Chemical Abstracts Service (CAS)-number and canonical simplified molecular-input line-entry system (SMILES) as identifier. Supplier, batch/LOT, purity, if available, and the selection reason are listed. (n.d.: not data)

No	Tc	IUPAC name/name	CAS-No.	Canonical SMILES	Supplier	Catalog No.–Batch No.	Purity	Selection reason
1	1.00	[(E)-2-nitroethenyl]benzene	102-96-5/5153-67-3	C1=CC=C(C=C1)C=C[N+](=O)[O-]	Merck,	8.18165–S37136	≥ 98%	Lead-structure
2	0.88	2-Nitroethylbenzene	6125-24-2	C1=CC=C(C=C1)CC[N+](=O)[O-]	Combi-Blocks, Inc	QC-3967–A77186	95%	w/o double bound
3	0.77	1-Nitro-2-[(E)-2-nitroethenyl]benzene	3156-39-6/670-67-7	C1=CC=C(C(=C1)C=C[N+](=O)[O-])[N+](=O)[O-]	abcr GmbH	AB179582–1405560	97%	High similarity
4	0.75	Styrene	100-42-5	C=CC1=CC=CC=C1	Sigma-Aldrich	45993–BCBX5031	≥ 98%	w/o nitro-group
5	0.74	2-Phenylethanamine	64-04-0	C1=CC=C(C=C1)CCN	Sigma-Aldrich	41346–BCCC2705	≥ 99%	Pro-mutagen
6	0.67	Nitrobenzene	98-95-3	C1=CC=C(C=C1)[N+](=O)[O-]	Sigma-Aldrich,	06084–BCCC2705	≥ 99%	w/o vinyl-group
7	0.64	(E)-3-phenylprop-2-enoic acid	140-10-3/621-82-9	C1=CC=C(C=C1)C=CC(=O)O	Sigma-Aldrich	C80857–MKCG1074	≥ 99%	-M control
8	0.59	5-[(E)-2-nitroethenyl]-1,3-benzodioxole	22568-48-5/1485-00-3	C1OC2=C(O1)C=C(C=C2)C=C[N+](=O)[O-]	Sigma-Aldrich	QW-1649–0001636156	n.d	Mostly positively predicted
9	0.58	2-Methoxy-5-[(E)-2-nitroethenyl]phenol	39816-35-8	COC1=C(C=C(C=C1)C=C[N+](=O)[O-])O	Combi-Blocks, Inc	S352918–A58409	95%	Mostly negatively predicted

Grouping criteria for these compounds are listed in Table 1: Compound no. 2, 2-nitroethylbenzene, lacks the double bond in the nitrovinyl moiety. Compound no. 3, 1-nitro-2-[(E)-2-nitroethenyl]benzene is overall highly similar to β-nitrostyrene. Compound no. 4, styrene, lacks the β-nitrostyrene-specific nitro-group. Compound no. 5, 2-phenylethanamine, is known to possess pro-mutagenic properties. Compound no. 6, nitrobenzene, contains no vinyl-group. Like β-nitrostyrene, compound no. 7, (E)-3-phenylprop-2-enoic acid, contains a -M- effect, but the nitro function is replaced by a carboxyl group. The last two compounds were chosen based on their QSAR alerts. Prediction for compound no. 8, 5-[(E)-2-nitroethenyl]-1,3-benzodioxole, indicated a potential mutagenic activity, whereas prediction for compound no. 9, 2-methoxy-5-[(E)-2-nitroethenyl]phenol, pointed mainly towards non-mutagenicity.

#### Technical reference compounds

Etoposide (Merck Millipore, USA 34105, Lot: 3202355, Purity: > 97%) dissolved in DMSO was used as positive control for both the alkaline comet assay and γH2A.X quantification, due to its ability to induce DNA double strand breaks. Colcemid (Merck Millipore, USA, L6221 Lot: 090F) was used from a ready to use stock solution (10 µg/mL) as an aneugenic positive control for quantification of pH3 (S10). Methyl methanesulfonate (MMS, 129925-5G, Lot: MKCM5645, Merck KGaA, Germany, Purity: 99.7%) diluted in cell culture medium, served as clastogenic positive control in the mouse lymphoma assay (MLA). Dimethyl sulfoxide (DMSO, Sigma-Aldrich, USA, 276855, Lot: STBJ4176, Purity: ≥ 99.9%) was chosen as a vehicle for all test compounds and subsequently as vehicle control. To lyse cells, 1% (v/v) Triton X-100 (AppliChem, Germany) was used during the cytotoxicity investigations.

### Cell models

#### L5178Y/TK^±^ mouse lymphoma cells (clone 3.7.2C)

L5178Y/TK^±^ mouse lymphoma cells (clone 3.7.2C, originally received from Boehringer Ingelheim Pharma GmbH & Co., Germany) were used to assess both cytotoxicity, clastogenicity and mutagenicity. Cell characteristics, cell culture and the cleansing procedure needed for the MLA were consistent with OECD Guideline No. 490.

Cells were propagated in 75 cm^2^ culture flasks at 37 °C in an incubator with a water saturated atmosphere, containing 5% CO_2_. The standard growth medium consisted of HEPES-buffered RPMI 1640 medium (Gibco Life Technologies, USA) supplemented with 2 mM L-glutamine and 10% heat-inactivated horse serum (Gibco Life Technologies, USA), 1 mM sodium pyruvate (Gibco Life Technologies), as well as 100 µg/mL streptomycin sulphate and 100 U/mL penicillin G sodium salt (Biochrom, UK). For subculturing, exponentially growing L5178Y/TK^±^ cells were centrifuged (5 min, 140×g), resuspended in cell culture medium, counted, and diluted as required (5 × 10^5^ L5178Y/TK^±^ mouse lymphoma cells in 20 mL cell culture medium).

#### WS1 cells

WS1 cells (LGC Standards, UK) are primary human skin fibroblasts and were used in the present study to assess membrane integrity and metabolic competence, as indicators for cytotoxicity, and to quantify pH3 (S10) and γH2A.X. WS1 cells were shown to be more suited for quantification of pH3 (S10) and γH2A.X than L5178Y/TK^±^ cells. For reasons see Sections “Cytotoxicity” and “Characterization of in vitro cytotoxicity”.

Cells were propagated in 75 cm^2^ culture flasks at 37 °C in an incubator with a water saturated atmosphere, containing 5% CO_2_. Eagle’s Minimum Essential Medium (EMEM, ATCC, USA) supplemented with 10% fetal bovine serum (Biochrom, UK) and 0.01% gentamicin (Gibco Life Technologies) served as cell culture medium. Cell were splitted by removing the cell culture supernatant, followed by a washing step with phosphate-buffered saline (PBS), a 3 min trypsinization step (0.05% Trypsin, 0.02% EDTA in PBS, PAN-Biotech GmbH, Germany) at 37 °C, resuspension in fresh cell culture medium, and final counting and dilution of the cell suspension, as required (10^6^ WS1 cells in 15 mL cell culture medium for a growth period of 4 days).

### Cytotoxicity

#### Membrane integrity—lactate dehydrogenase (LDH) release

Prior to treatment, 5 × 10^4^ WS1 cells per well were seeded in 1 mL cell culture medium into 24 well plates (Thermo Fisher Scientific Inc., USA), and preincubated for 24 h. L5178Y/TK^±^ cells were diluted directly before treatment in cell culture medium to a cell density of 5 × 10^5^ cells per mL, from which 0.5 mL were placed into 1.5 mL reaction cups (Eppendorf SE, Germany). Subsequent treatments were started by adding the respective volume of freshly prepared 500-fold concentrated stock solutions in DMSO to the wells/reaction cups. Final reaction volumes amounted to 1 mL and 0.5 mL for the WS1 and L5178Y/TK^±^ cells, respectively. After 4 h of incubation (L5178Y/TK^±^ cells were incubated under shaking at 300 rpm and subsequently pelleted by centrifugation at 140×g for 5 min), 50 µL of cell supernatants were removed and mixed with the same volume of freshly prepared LDH-reagent (Cytotoxicity Detection Kit, Hoffmann-La Roche, Switzerland). The experiments were performed in triplicates (biological replicates). After 30 min of incubation in the dark at room temperature, each sample was measured in triplicate using a SPECTRAmax microplate reader (Molecular Devices, Germany; λ_measurement_ = 490 nm, λ_reference_ = 630 nm).

As technical positive control (100% cytotoxicity), cells were incubated with a final concentration of 1% (v/v) Triton X-100, 10 min prior the end of the treatment phase. Untreated vehicle control cells were set to 0% cytotoxicity. DMSO-treated cells were used as vehicle control and thus for statistical comparisons. Relative cytotoxicity was determined as follows: ([treatment-vehicle control]/[positive control − vehicle control)]*100.

#### Metabolic competence—water soluble tetrazolium salt (WST-1)

After incubation of WS1 cells with the test and reference compounds, incubation media were replaced by freshly prepared cell culture medium, containing 10% (v/v) of WST-1 reagent (Hoffmann-La Roche, Switzerland). In the case of L5178Y/TK^±^ cells, cells were pelleted by centrifugation (140×g, 5 min), supernatant was discarded, and cells resuspended in freshly prepared cell culture medium, supplemented with 10% (v/v) WST-1 reagent, followed by a short vortexing step. After 60 min of incubation with WST-1 stain at 37 °C and 5% CO_2_ in a water-saturated atmosphere, 100 µL of supernatant was captured per sample (for L5178Y/TK^±^ cells after pelleting) and each sample measured in triplicate using a SPECTRAmax microplate reader (λ_measurement_ = 450 nm, λ_reference_ = 630 nm).

To get positive controls (0% viability) cells were incubated with a final concentration of 1% (v/v) Triton X-100 for 10 min prior to the end of treatment. Untreated vehicle control cells were set to 100% viability. The DMSO-treated vehicle control cells were used for statistical comparison. Cell viability ass determined as follows: 100-([vehicle control − treatment]/[vehicle control − positive control])*100.

### Genotoxicity

#### DNA-strand breaks—in vitro alkaline comet assay (comet assay)

The comet assay was performed, as described previously by Volk et al. (2018). In brief, L5178Y/TK^±^ cells (2 × 10^5^ per sample) were incubated for 1 h with β-nitrostyrene or the respective reference compounds. Prior treatment, glass slides with one roughened side (Marienfeld GmbH & Co.KG, Germany) were pre-coated with 40 µL of 0.5% normal melting agarose (NMA, Sigma, Germany). Agarose was quickly distributed over the slide surface and subsequently dried at 37 °C. Another 100 µL of NMA was added, covered with a cover slip (Marienfeld GmbH & Co.KG) and stored at 4 °C in a refrigerator. After cell treatment, cells were centrifuged (140×g, 5 min), the supernatants discarded, the pellet gently resuspended in 80 µL of 0.75% low-melting agarose (LMA, Sigma; pre-heated to 37 °C), and quickly spread over the surface of an NMA pre-coated glass slide by adding a cover slip. Slides were then stored at 4 °C for agarose polymerization, with subsequent addition of a second NMA layer (100 µL). After the last polymerization step slides were submerged in lysis solution (2.5 M NaCl, 0.1 M Na_2_EDTA, 10 mM Tris–HCl, 8 g/L NaOH, 1% Triton-X100, 10% DMSO) and stored at 4 °C. The next day, slides were placed into an ice-cooled electrophoresis chamber and submerged in electrophoresis buffer (300 mM NaOH, 1 mM Na_2_EDTA, pH > 13, 4 °C). After a 20 min DNA unwinding step electrophoresis was performed for 20 min at 1 V/cm and 300 mA. Thereafter, slides were neutralized using Tris-buffer (0.4 M Tris–HCl, pH 7.4) and stained with 80 µL of ethidium bromide (0,002%; Merck KGaA) per slide. A fluorescence microscope (Axioskop, Carl Zeiss, Germany) and the Comet Assay III Software (Perceptive Instruments, UK) were used for slide analysis. Tail intensity (TI), defined as the percentage of DNA in the comet tail, served as the main and most accepted endpoint for comet assays (see OECD Guideline No. 489), and 100 nuclei were assessed per slide.

#### γH2A.X and pH3 (S10) quantification—immunofluorescence

Following the protocol of Reamon-Buettner et al. (2021), 5 × 10^4^ WS1 cells were seeded in 200 µL of medium on 18 mm-cover slips (VWR International, USA) and placed into 12 well plates (Thermo Fisher Scientific Inc.). After 2 h of incubation at 37 °C and 5% CO_2_ in a water-saturated atmosphere, wells were supplied with additional 1.8 mL of preheated cell culture medium and incubated overnight. After medium exchange, test compounds were added from freshly prepared 500-fold DMSO stock solutions. After 4 (γH2AX) or 24 h [pH3 (S10)] of incubation, cells were placed on ice and fixed by replacing the medium with − 20 °C-cold methanol. After 10 min of incubation at − 20 °C, cells were washed once with 1 mL of PBS. For permeabilization, cells were incubated with 1 mL of 0.5% Triton X-100 (v/v) in PBS for 5 min. Cells were blocked for 30 min in 1 mL 1% bovine serum albumin (BSA) (w/v) in Tris-buffered solution (TBS). For pH3 (S10) staining, cells were incubated with a final concentration of 2 ng/mL of the primary monoclonal mouse anti-pH3 (S10) antibody (abcam, ab14955, UK) in 250 µL of 0.1% BSA/TBS. For γH2A.X staining, cells were incubated with a final concentration of 0.98 ng/mL primary monoclonal rabbit anti-γH2A.X S139 antibody (ab81299, abcam) in 500 µL of 0.1% BSA/TBS. After an overnight incubation at 4 °C, cells were washed three times with PBS and incubated with a final concentration of 5 ng/mL of the respective secondary antibody [pH3 (S10): anti-mouse IgG Alexa Fluor® 568, ab175473, abcam; γH2A.X: anti-rabbit IgG Alexa Fluor® 488, ab150077, abcam] in 500 µL of 0.1% BSA/TBS for 90 min in the dark. Finally, cells were counterstained with 4′,6-Diamidin-2-phenylindol (DAPI, Merck KGaA) in a final concentration of 0.25 µg/mL in distilled water. After a washing step with PBS, cover slips were mounted in an upside-down manner on glass slide (Polysine slide, Menzel Gläser, Germany) using Vectashield H-1000 (VECTOR LABORATORIES INC., USA). Mounted cover slips were stored at 4 °C in the dark until analysis. Endpoint analysis was done with a Metafer5 System (Version 3.13.5, MetaSystems, Germany). Slides were analyzed individually in a multiparametric manner for quantification of predefined cell populations, which are described in detail in section S7.1. For γH2A.X at least 2000 cells were analyzed, and for the pH3 (S10) at least 5000 cell nuclei.

#### Mutagenicity—mouse lymphoma assay (MLA)

To look for mutagenicity of the lead compound β-nitrostyrene, an MLA was performed, as described previously by Volk et al. (2018) and based on the respective OECD Guideline (OECD 2016b). Cell aliquots of 1 × 10^7^ L5178Y/TK^±^ cells per treatment condition in 20 mL horse serum-reduced (5%; RPMI-5) cell culture medium were exposed for 4h without S9-mix to four different concentrations of β-nitrostyrene (no. 1; 0.5 µM–5 µM), to 0.2% DMSO as vehicle control, or to 5 µL/mL MMS as clastogenic positive control. Subsequently, cells were washed twice in 20 ml cell culture medium by pelleting (140×g, 5 min) and finally resuspended in 10 mL of cell culture medium with 10% horse serum (RPMI-10). Cells were counted (cell setup_day0_; CASY Cell Counter, Schärfe System GmbH, Germany) and adjusted to a cell concentration of 2 × 10^5^ cell/mL to prepare survivor 1 plates per treatment (evauation of plating efficiency) and subculture 1 flasks (phenotypic expression prior to mutant selection). Survivor 1 plates were generated by seeding 1.6/cell per 200 µL into each well of two 96-well plates using cell culture medium supplemented with 20% horse serum (RPMI-20). These plates were cultured for 7 days to determine the plating efficiency after treatment, by counting the number of empty wells. Furthermore, subculture 1 was prepared, based on the previously prepared 2 × 10^5^ cell/mL cell suspension in RPMI-10. Approximately 6 × 10^6^ cells in 30 ml or the remaining cells, if less, were placed in cell culture flasks incubated in a cell incubator at 37 °C. After 24 h, subculture 1 was centrifuged, the cell pellet resuspended in RPMI-10 and cells counted (cell count_day1_). Subsequently, 6 × 10^6^ cells per flask, or the remaining cells (cell setup_day1_), were again seeded into two flasks in a volume of 30 mL per flask to generate subculture 2.

After 24 h of incubation, cells of subculture 2 were pelleted (140×g, 5 min), counted (cell count_day2_) and adjusted to 1 × 10^4^ cells/mL in RPMI-20 (cell setup_day2_). Two survivor 2 plates were prepared from subculture 2 by plating 1.6 cells/well in 200 µL per well of two 96-well plates. After 7 days plating efficiency of the survivor 2 plates were assessed by counting the no. of empty wells. Additionally, subculture 2 was used for the TFT-selection plates to determine mutation frequency. TFT-selection plates were prepared by adding 850 µl of a TFT–stock solution (300 µg/mL trifluorothymidine [TFT; Sigma–Aldrich] in aqua dest.) to 85 ml of the diluted cell suspension in RPMI-20 (1 × 10^4^ cells/mL). Subsequently, 200 µL (1 × 10^3^ cells) were plated per well of four 96-well plates per treatment. After 11 days of incubations, small and large grown colonies were counted.

Suspension growth (SG), as the measure for cytotoxicity of the test compound, was calculated as follows: (cell count_day1_/cell setup_day0_) × (cell count_day2_/cell setup_day1_). To normalize the results against the control treatment, the relative suspension growth (RSG) = (SG_treatment_/SG_control_) was determined. The cloning efficiency (CE) was assessed using: CE =  − ln(no. of empty wells/no. of total wells)/2000. The CE values were compared to the control treatment yielding the relative cloning efficiency (RCE) = CE_treatment_/CE_control_. Finally, the relative total growth (RTG) was calculated as following: RTG = RSG × RCE × 100. The mutation frequency (MF) was calculated using: MF = (CE_TFT selection_/CE_survivor 2_) × 10^6^.

### Statistical analysis

Despite the recommendations given in the OECD test guideline No. 489 for evaluation of alkaline comet assay data, the arithmetic mean was chosen as summarizing measure per slide instead of the median, due to a more bimodal data distribution of TI values, based on the observation that β-nitrostyrene seemed to preferentially affect cells going through cell cycle at the time of treatment. In the case of a bimodal data distributions the median might underestimate effects, depending on the size of the two subpopulations, with the fraction of non-dividing cells with no or low damage being bigger in size in the present study. Irrespective of potential overestimation of DNA damage, the arithmetic mean was finally used as summarizing measure, due to precautionary reasons.

Statistical evaluation of cytotoxicity and genotoxicity data was done by One-Way Analysis of Variance (ANOVA) with Dunnett’s post hoc testing using the GraphPad Prism software (version 10.1.2, GraphPad Software, Lnc. USA). One-Way ANOVA with Dunnett’s post hoc testing was chosen to respect alpha error inflation in testing more than one treatment against the same vehicle control. Additionally, this test is robust against potential non-normality of data and low number of data points. Effects were judged as significantly different from respective vehicle controls, if *p* ≤ 0.05.

The MLA was performed in a single experiment in duplicate, as foreseen by OECD 490, with finally four plates per concentration analyzed for wells with big and small colonies and calculation of MF. An increase in MF was considered biologically relevant, if it exceeded the mean value MF of the vehicle control by the “Global Evaluation Factor” of 126 × 10^–6^ (Moore et al. 2006).

## Results

Initially, in silico characterization was performed to select β-nitrostyrene derivatives for the in vitro investigations, with the aim to identify the structural elements forming the basis for its adverse biological reactivity.

### In silico characterization of the compound group

As stated in Section “In silico characterization of the compound group”, in total 29 β-nitrostyrene derivatives were identified. Nine meaningful test compounds were selected in a grouping approach for an in-depth in silico and in vitro characterization. The selection criteria are described in Section “Characterization of in vitro clastogenicity”.

#### Tanimoto similarity

**Fig. 1:**
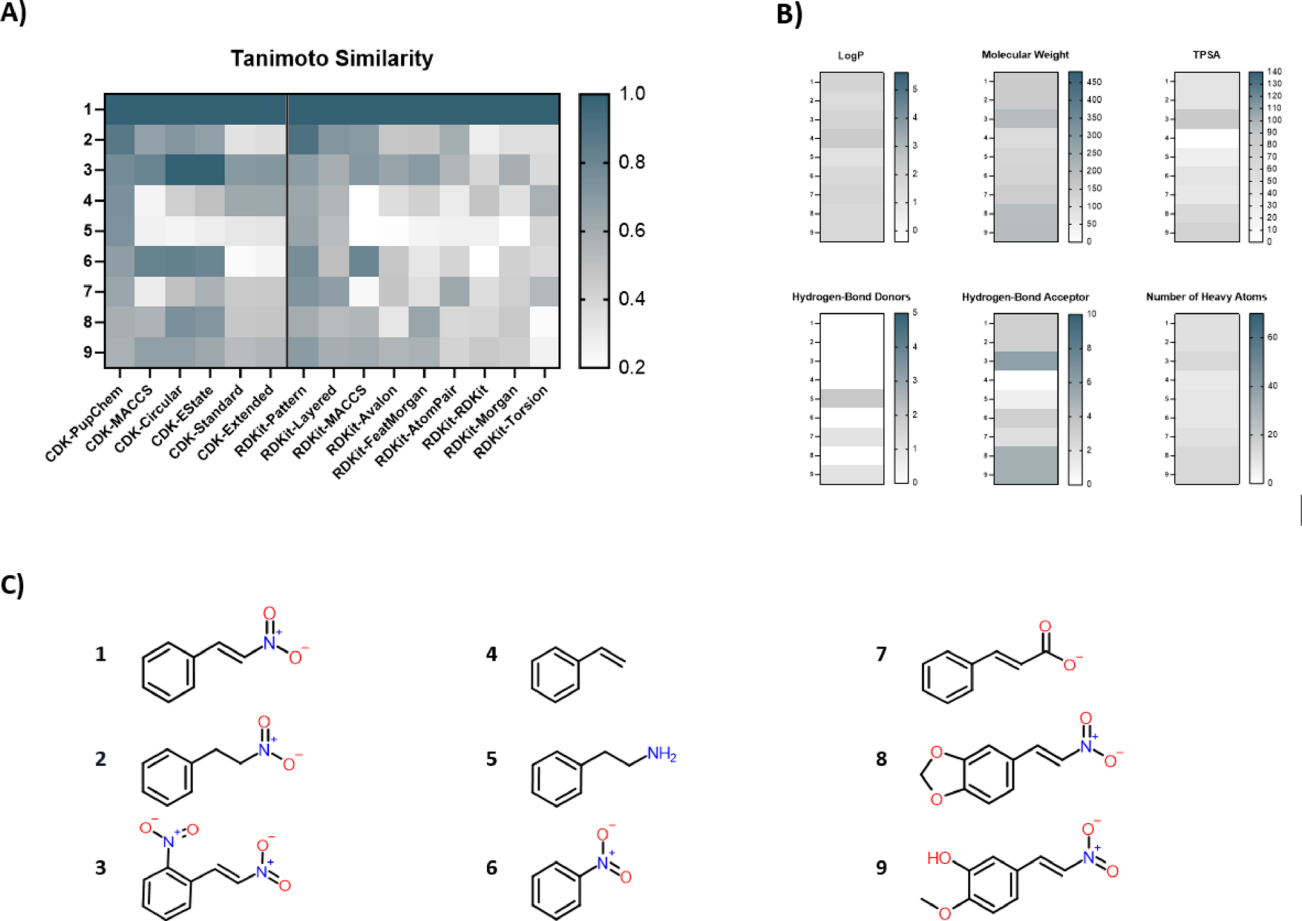
In silico characterization of β-nitrostyrene derivatives. **A** Tanimoto coefficients computed with different fingerprints. The PubChem fingerprint was utilized for sorting. Blue indicates a high similarity to β-nitrostyrene, whereas white reflects low similarity (below 0.2). **B** Chemical descriptors based on the rule of five. The color scheme reflects the refined descriptor range of the Lipinski rule of five (Ghose et al. 1999). **C** Structures of the nine test compounds. Abbreviation: octanol–water partition coefficient (LogP), topological polar surface area (TPSA)

Structural similarities were assessed using various molecular fingerprints (see Fig. 1A for test compounds and Fig. S2 for the complete dataset). The PubChem fingerprint, used for final compound sorting, yielded similarity values between 0.877 and 0.578. The highest similarity scores, when comparing the fingerprints, were obtained by using the RDKit-Pattern fingerprint, while the lowest were computed by the RDKit-RDKit fingerprint. Among the test compounds, compound no. 3 was identified as the overall most similar structure, compared to β-nitrostyrene, based on eight fingerprints (mean similarity-value: 0.68), whereas compound no. 5 showed the lowest values according to seven fingerprints (mean similarity-value: 0.34).

#### Chemical descriptors

To check for compound-category homogeneity, various chemical descriptors were assessed (see Fig. 1B and Fig. S3). The entire dataset aligns with the Lipinski rule of five, which indicates good oral bioavailability (Lipinski et al. 1997; Ghose et al. 1999). For example, LogP values range from 1.19 to 2.33. Furthermore, TPSA values between 0 and 86.28 were reported. However, the number of heavy atoms is below the recommended minimum of 20 for drug like compounds, as specified by Ghose et al. (1999), with the test compounds containing between 8 and 14 heavy atoms.

#### QSAR Ames test mutagenicity predictions

Ames test-based mutagenicity alerts were calculated using three QSAR algorithms, i.e., GT1_BMUT, PHARM_BMUT, and GT_EXPERT. For all 29 compounds, the alerts of these algorithms are sown in Table S4 to S6. None of the nine chosen test compounds was found to be out of domain, as shown in Table 2. As mentioned in this table, the predictions were complemented by literature results to proof the made predictions. In general, the alerts were rather homogeneous for each compound, which can be underpinned by the fact that three of the nine compounds were known by all three algorithms used. However, opposing alerts were generated for compounds no. 1 and 8.

**Table 2:** QASR alerts for Ames test-based mutagenicity algorithms. These results were complemented by literature reports

No	GT1_BMUT	PHARM_BMUT	GT_EXPERT	Genotoxicity and cytotoxicity literature results
1	Known Negative	Positive	Known Negative	Weakly mutagenic in Ames test; TA 100 (Zeiger et al. 1992) Cytotoxic in vitro and antineoplastic in vivo (Doré and Viel 1975) Pro-apoptotic and cytotoxic in rat pituitary GH_3_ tumor cells (Kaap et al. 2003) Pro-apoptotic effect in neutrophil progenitor cells (Bartels et al. 2014) Cytotoxic in LoVo cells (Werner et al. 2007) Cytotoxicity in HeLa cells (Renu Mohan et al. 2006)
2	Inconclusive	Negative	Negative	Not cytotoxic in Burkitt’s lymphoma cells (Andrew J. Byrne et al. 2018) Not cytotoxic in HeLa cells (Salillas et al. 2019) Not cytotoxic in mouse fibroblasts (Ugheighele et al. 2020) Not genotoxic in vivo (Oyeniyi et al. 2022) Cytotoxic in vitro (Doré and Viel 1975)
3	Inconclusive	Positive	Positive	Cytotoxic in rat pituitary GH3 tumor cells (Kaap et al. 2003) Pro-apoptotic in neutrophil progenitor cells (Bartels et al. 2014) Cytotoxic in vitro (Doré and Viel 1975) Cytotoxicity in HeLa cells (Renu Mohan et al. 2006)
4	Known Positive	Known Positive	Known Positive	No direct mutagenic effect in Ames test; A 98, TA 100, TA 1535, TA 1537, TA 1538 (Meester et al. 1977) Not cytotoxic without metabolic activation in cHo1 cells (Chung et al. 2006) Not cytotoxic in vitro (Doré and Viel 1975)
5	Negative	Negative	Negative	Not mutagen in *A. thaliana* (Gichner and Velemínský, 1984) Not mutagen in Ames test; TA 100 (Laires et al. 1993) Pro-mutagen in Ames test; TA 1535 (Fiala et al. 1981) No significant toxicity in HeLa cells (Salillas et al. 2019)
6	Known Negative	Known Negative	Known Negative	Not mutagen in Ames test; TA 98 and TA 100 (Aßmann et al. 1997) No genotoxic effect without metabolic activation in various bacterial and mammalian test system (Davies 2003)
7	Known Negative	Known Negative	Known Negative	Not cytotoxic in various cell lines (Kaap et al. 2003; Lee et al. 2004) Non genotoxic in human peripheral blood lymphocytes (Taner et al. 2017) Not cytotoxic in rat pituitary GH3 tumor cells (Kaap et al. 2003)
8	Positive	Positive	Negative	Pro-apoptotic effect in pancreatic cancer cells (Liu et al. 2020) Cytotoxic in MDA-MB-231 and HBL-100 cell (Hsieh et al. 2010) Cytotoxic in HeLa cells (Renu Mohan et al. 2006)
9	Inconclusive	Negative	Negative	Cytotoxic and genotoxic and pro-apoptotic properties in various cancer cell lines (Chiu et al. 2016; Hung et al. 2016; Tsai et al. 2017a, 2017b; Wang et al. 2017b, 2017a) Cytotoxic in MDA-MB-231 and HBL-100 cell (Hsieh et al. 2010)

### Characterization of in vitro cytotoxicity

**Fig. 2:**
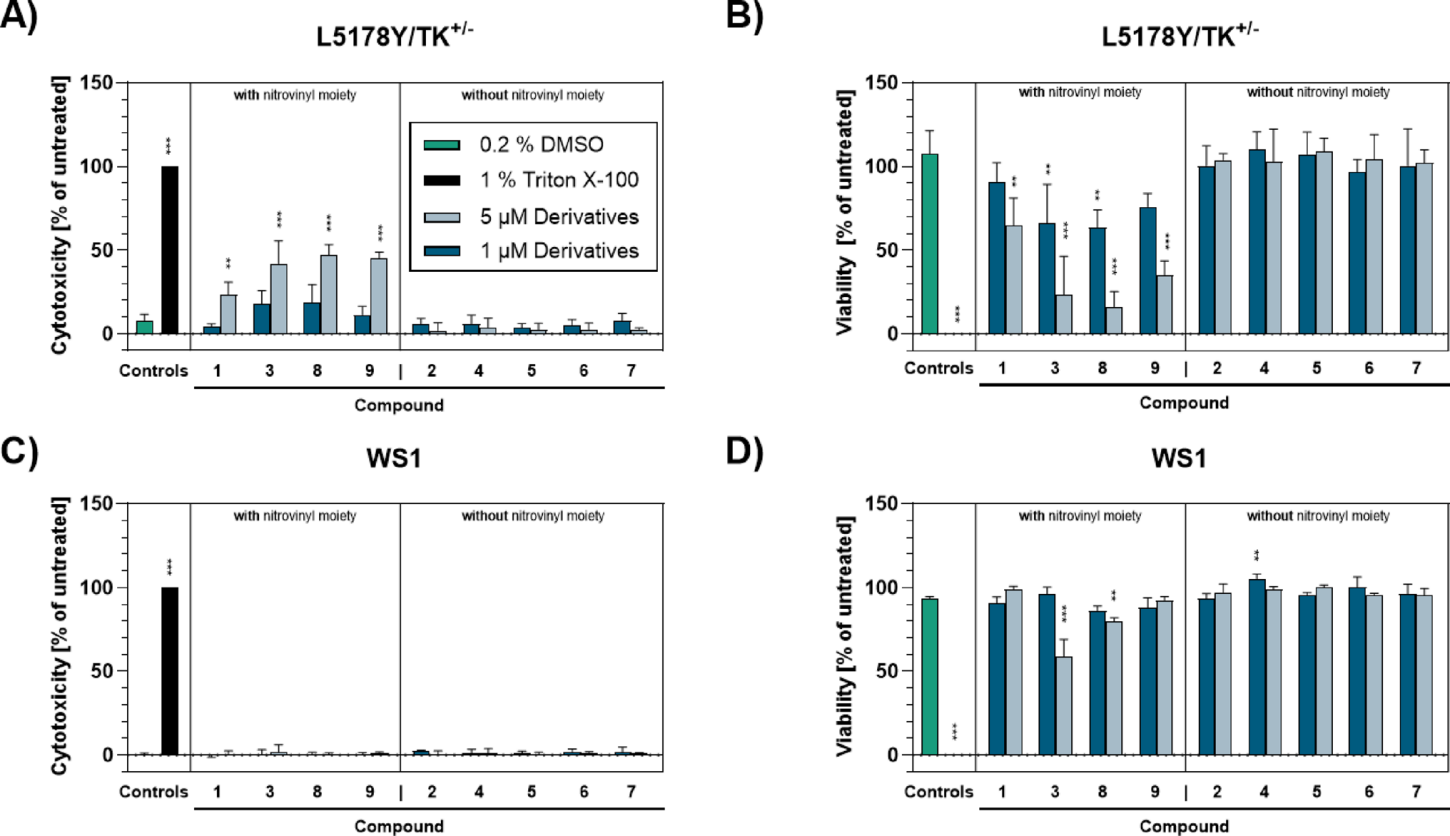
Cytotoxicity assessment of β-nitrostyrene and its selected derivatives at 1 and 5 µM (dark and light blue bars, respectively). Results were generated in L5178Y/TK^±^ cells (**A** and** B**) or in WS1 cells (**C** and **D**). Membrane integrity was measured using lactate dehydrogenase (LDH) liberation as endpoint (see **A** and **C**). Metabolic competence was analyzed by measurement of water-soluble tetrazolium salt 1 (WST-1) turnover (see **B** and **D**). For all experiments, 0.2% DMSO treated cells (green bars) served as vehicle controls, and cells lysed with 1% Triton X-100 (black bars) as positive controls. Data represent means ± SD of n = 3 independent cell cultures. Statistically significantly different from DMSO-treated cells: **p ≤ 0.01, ***p ≤ 0.001, One-Way ANOVA with Dunnett’s post-hoc test

Both, cytotoxicity and genotoxicity are modes of actions of anti-neoplastic drugs, and often interconnected (Vock et al. 1998) Therefore, both endpoints were taken into account in this study. For better comparability both cyto- and genotoxicity testing was performed with the same cell lines, i.e., L5178Y/TK^±^ (Fig. 2A, B) and WS1 cells (Fig. 2C, D) after 4 h of incubation. As cytotoxicity endpoints membrane integrity was measured by LDH release, as depicted in Fig. 2A, C, and metabolic activity was analyzed by WST-1 turnover, and given in Fig. 2B, D.

In either cell line, compounds lacking the nitrovinyl moiety (compounds no. 2, 4, 5, 6, 7) did not induce a significant cytotoxic effect in the concentrations tested. In contrast, compounds harboring a nitrovinyl moiety (compounds no. 1, 3, 8, 9) mediated a significant cytotoxic effect, which was overall more pronounced in L5178Y/TK^±^ cells than in WS1 cells. The maximum effect regarding LDH release was mediated by compound no. 8 in L5178Y/TK^±^ cells, amounting to 47.2 ± 5.28% cytotoxicity, whereas DMSO treated cells showed a cytotoxicity of 8.0 ± 3.19%. This compound also reduced metabolic activity and thus viability of L5178Y/TK^±^ cells to 16.2 ± 7.47%, opposing the 107.5 ± 11.44% viability of DMSO-treated cells. Compounds no. 3 and 8 exhibited comparable cytotoxic effects, with compound no. 3 inducing more pronounced effects in L5178Y/TK^±^ cells than in WS1 cells, whereas compound no. 8 was more cytotoxic in WS1 cells. Therefore, by considering both cell lines the potency of cytotoxic effects of the investigated chemical structures could be ranked as follows: no. 3 = no. 8 > no. 9 > no. 1.

### Characterization of in vitro clastogenicity

**Fig. 3:**
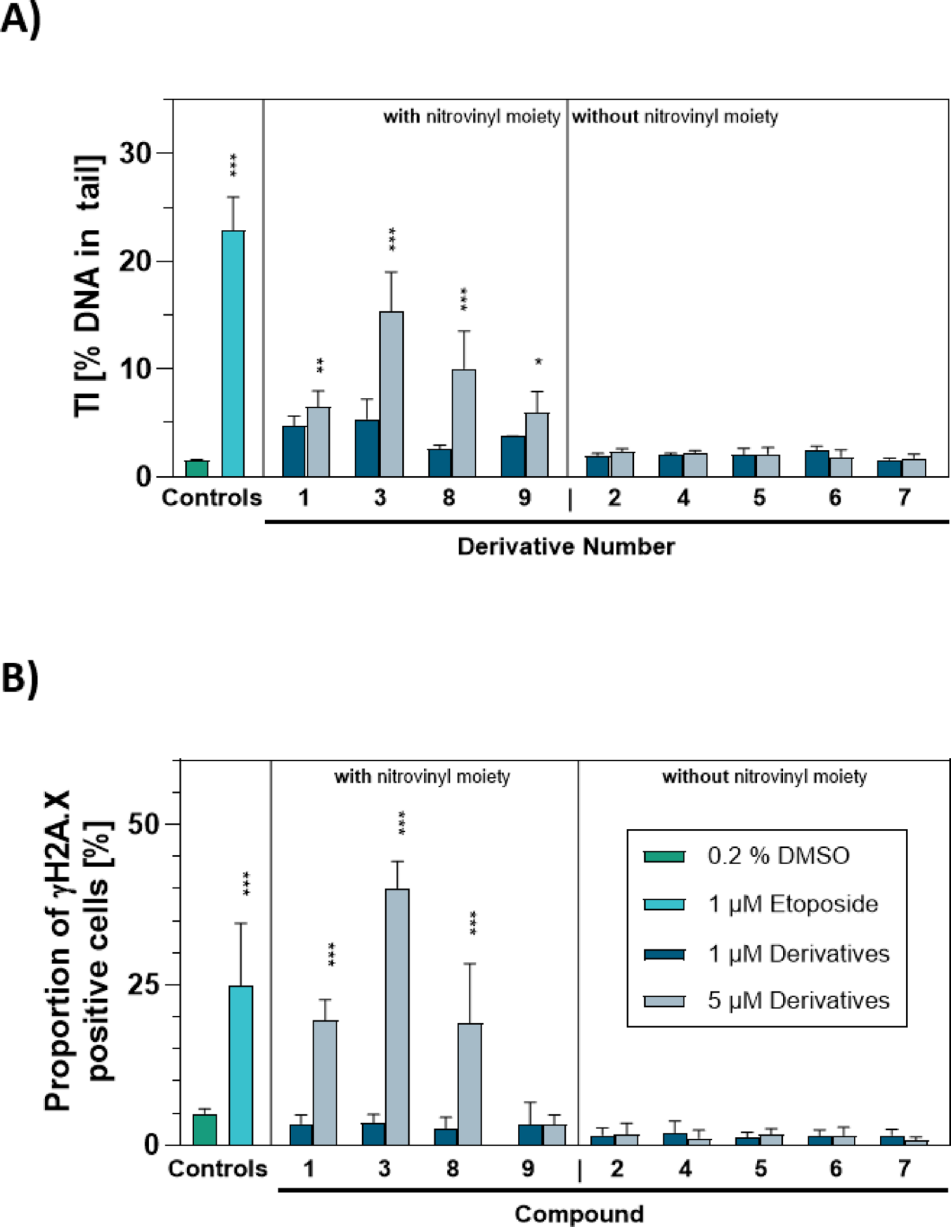
DNA damaging potential of β-nitrostyrene and its selected derivatives at 1 and 5 µM (dark and light blue bars respectively). **A** The induction of DNA damage was measured after 1 h of incubation in an in vitro alkaline comet assay with L5178Y/TK^±^ cells. **B** Depicted are the proportions of γH2A.X positive WS1 cells, calculated from analysis of at least 2000 nuclei. For both experiments, 0.2% DMSO-treated cells (green bars) served as vehicle control, and cell treated with 1 µM Etoposide (turquoise bars) served as genotoxic positive control. Data in both investigations represent means ± SD of n = 3 independent cell cultures. Statistically significant different from DMSO-treated cells: *p ≤ 0.05, **p ≤ 0.01, ***p ≤ 0.001, One-Way ANOVA with Dunnett’s post-hoc test

To assess the clastogenic potential of β-nitrostyrene and its derivatives, we conducted an alkaline comet assay, able to detect both DNA-single and -double strand breaks and alkali-labile sites. The experiment was performed in L5178Y/TK^±^ cells, which were incubated for 1 h with 1 and 5 µM of the test-compounds. In line with the results on cytotoxicity, statistically significant induction of DNA damage was limited to compounds possessing a nitrovinyl moiety. The strongest effect, as depicted in Fig. 3A, was induced by compound no. 3 with a mean TI of 15.3 ± 3.01%, whereas mean TI of the vehicle control amounted to 1.0 ± 0.08%. The results of the alkaline comet assay could be ranked as follows with regard to their DNA damaging potential: compound no. 3 > no. 8 > no. 1 = no. 9.

Furthermore, the phosphorylation of H2A.X, as a marker for amongst others DNA-double strand break induction, was quantified in WS1 cells. WS1 cells were chosen, as L5178Y/TK^±^ cells showed a γH2A.X-pan-staining phenotype after treatment, with no chance to quantify γH2A.X foci. WS1 cells, in contrast demonstrated the preferred γH2A.X foci phenotype (see Fig. S4). The proportions of γH2A.X-positive WS1 nuclei after 4 h of test-compound exposure are shown in Fig. 3B. Quantification was done using the semi-automated staining intensity classifier, implemented in the Metafer5 software, as given in the supplementary materials (Fig. S9) as well as representative stained cells sorted by staining intensity value (Fig. S5). In line with the comet assay data (Fig. 3A) and the cytotoxicity results in Section “Cytotoxicity and genotoxicity”, compound no. 3 induced the highest percentage of cells, classified as γH2A.X- positive (40.1 ± 4.40%), as compared to 4.9 ± 0.63% for the vehicle control. Unexpectedly, compound no. 9, which possesses a nitrovinyl moiety, did not significantly increase the proportion of γH2A.X- positive cells, compared to the vehicle control. Overall, the test compounds with a nitrovinyl moiety can be ranked with regard to their potential to induce γH2A.X foci and thus DNA-double strand breaks as follows: no. 3 > no. 8 > no. 1 > no. 9. All compounds without nitrovinyl moiety did not mediate induction of γH2A.X foci.

### Characterization of in vitro aneugenicity

**Fig. 4:**
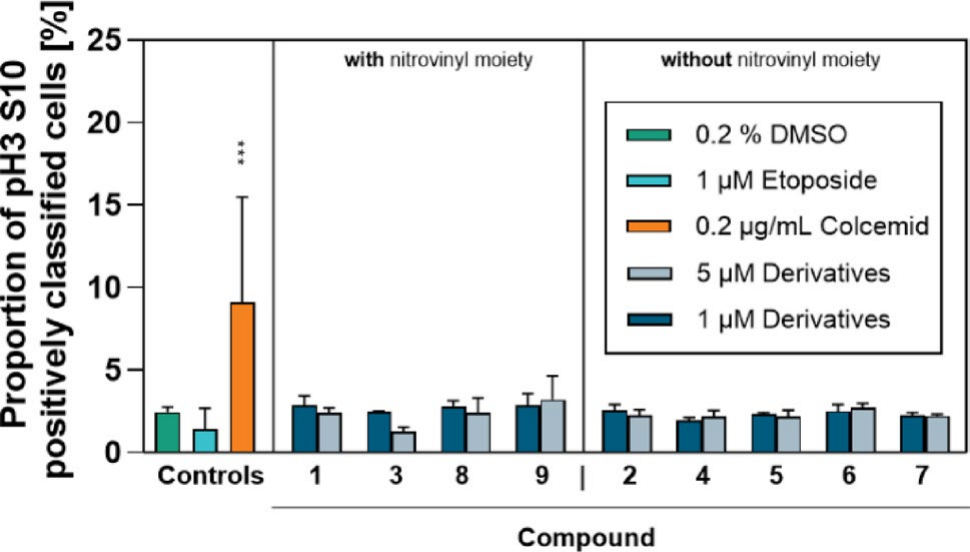
Assessment of the aneugenic potential of the different β-nitrostyrene derivatives in L5178Y/TK^±^ mouse lymphoma cells, using pH3 (S10) as mitotic arrest marker. Cells were incubated for 24 h with 1 (dark blue bars) or 5 µM (light blue bars) of the different compounds. At least 5000 nuclei were analyzed and proportions of pH3 (S10)-positive cells calculated. Cells incubated with 0.2% DMSO served as vehicle control (green bar). Cells treated with 1 µM Etoposide are (turquoise bar) and cells treated with 0.2 g/mL Colcemid (orange bar) were used as positive controls for clastogenicity and aneugenicity, respectively. Data represent means ± SD of n = 3 independent cell cultures. Statistically significantly different from DMSO-treated cells: ***p ≤ 0.001, ANOVA with Dunnett Dunnett’s post-hoc test

In addition, aneugenicity, as one central mode of action of anti-cancer drugs, was assessed using the mitosis marker pH3 (S10). WS1 cells were stained for pH3 (S10) expression after 24 h of test compound exposure. Stained cells nuclei were quantified using the semi-automated Metafer5 System and a classifier comprising two different elements, staining intensity and area parameter as described in the supplementary materials (Fig. S10). Representative stained cells are displayed in Fig. S6 and Fig. S7 sorted by staining intensity and area parameter value, respectively. To ensure a positive classification across all phases of mitoses, these are representatively given in Fig. S8. The resulting proportion of pH3 (S10)-positive WS1 cell nuclei are shown in Fig. 4.

None of the test compounds significantly increased or decreased the proportion of pH3 (S10)-positive cell nuclei, as compared to the vehicle control [2.4 ± 0.25% pH3 (S10) positive cells]. As a proof of concept, Colcemid (0.2 µg/mL), as a positive control for aneugenicity, but not Etoposide, as an inducer DNA strand breaks, statistically significantly increased the proportion of pH3 (S10)-positive cell nuclei.

### Characterization of in vitro mutagenicity of β-nitrostyrene

The mutagenic potential of the lead structure β-nitrostyrene (compound no. 1) was evaluated using a mouse lymphoma assay, based on the respective OECD guideline No. 490 (OECD 2016b). The mutation frequencies (MF) of four β-nitrostyrene concentrations (0.5–5 µM) as well as of the positive control MMS (5 µM) are shown in Table 3. The increase in MF detected at the highest β-nitrostyrene concentration of 5 µM with 1229.5 × 10^–6^ was considered biologically relevant as its MF value exceeded the MF of the vehicle control, which was 201.5 × 10^–6^ by at least 126 × 10^–6^ (Moore et al. 2006). At the “positive” concentration of 5 µM, the MF of small colonies was rather high (633.4 × 10^–6^), indicating induction of structural chromosomal aberrations, which was in line with the above reported clastogenic potential of β-nitrostyrene, as determined by the alkaline comet assay and quantification of γH2A.X-positive cell nuclei.

**Table 3:** Mutagenicity and cytotoxicity of β-nitrostyrene, as assessed in the mouse lymphoma assay (MLA). As suggested by OECD No. 490 L5178Y/TK^±^ mouse lymphoma cells were treated with four different β-nitrostyrene (compound no. 1) concentrations (0.5–5 µM) for 4 h and then washed and further processed. The mutagenic potential was quantified as calculated mutation frequencies (MF). Cytotoxicity was demonstrated by reduction in suspension growth (SG), relative total growth (RTG) and cloning efficiency (CE). Data represents means, gathered from one experiment performed in duplicates. MMS served as mutagenic positive and 0.2% DMSO as vehicle control. Validity criteria according to OECD No. 490 are given in the notes

Treatment	MF^a^	relevance:	MF [10^–6^]	MF [10^–6^]	small	RTG^b^	SG^c^	CE^d^
	[10^–6^]	untreated + 126	small colonies	large colonies	colonies [%]	[%]	Suvivor 2	[%]
0 µM/0.2% DMSO	201.5	no	32.8	165.6	18.0	95.53	12.89	83
0.5 µM β-nitrostyrene	232.9	no	81.3	140.9	37.6	72.73	10.87	75
1 µM β-nitrostyrene	165.7	no	67.7	92.3	42.9	74.69	9.07	92
2.5 µM β-nitrostyrene	281.5	no	97.2	167.2	38.0	63.67	9.52	75
5 µM β-nitrostyrene	1229.5	yes	633.4	288.6	64.9	22.77	4.65	55
59 µM MMS	817	yes	430.9	273.4	59.0	58.79	10.42	63

Validity criteria according to OECD No. 490:

^a^MF: acceptability range for vehicle control: 50–170 × 10^–6^; pos. control: absolute MF: 300 × 10^–6^; small colonies: 40%, 150 × 10^–6^

^b^RTG (measure for cytotoxicity): pos. control: above 10%, highest concentration of test compound: 10–30%

^c^SG (measure for cell-number increase): acceptability range (4 h, neg. control): 8–32

^d^CE: acceptable range (neg. control): 65–120%

When applying the validity criteria laid down in OECD No. 490, the MF of the vehicle control (201.5 × 10^–6^), was slightly higher than the prescribed range of 50–170 × 10^–6^. The remaining criteria (relative total growth [RTG] above 10% for the positive control and within 10–30% for the highest concentration of the test compound; suspension growth [SG] in the range 8–32 of the vehicle control; cloning efficiency [CE] of the vehicle control in the range of 65–120%; MF of positive control 300 × 10^–6^ above negative control with at least 40% of the MF is rooted in the formation of small colonies) matched the requirements of OECD No. 490. Irrespective of the fact that the basal MF was slightly too high, the MLA was, therefore, regarded as valid, since the slightly elevated mutation frequency of the vehicle control seemed negligible, when considering the very high mutation frequency at the highest test compound concentration.

## Discussion

β-Nitrostyrene (Doré and Viel 1975) and its derivatives (Tsai et al. 2017a; Wang et al. 2017a; Panina et al. 2022; Byrne et al. 2023; McKeown et al. 2024) have been proposed as anti-neoplastic drug candidates. However, understanding of the underlying structural features, vital for their biological effects remain poorly understood, thus limiting therapeutic applicability. Therefore, we investigated nine meaningful β-nitrostyrene derivatives, chosen by a grouping-based in silico characterization. Subsequent in vitro characterization of their cytotoxicity revealed that derivatives possessing a nitrovinyl moiety exhibited strong adverse effects in WS1 and L5178Y/TK^±^ cells. Genotoxic effects were also exclusively seen with compounds harboring this functional group. However, induction of γH2A.X foci was not observed for all compounds with nitrovinyl moiety.

### Test-compound selection and in silico characterization

Out of the 29 compounds identified form literature reports, 9 test-compounds were selected with the focus on assessing the structure–activity relationship of the lead structure β-nitrostyrene. For the whole compound group the category homogeneity and drug likeness was tested using a subset of chemical descriptors, namely the rule of five by Lipinski et al. (1997), and no violations were detected. According to Ghose et al. (1999), the number of heavy atoms of a drug are expected to be between 20 and 70 atoms, which was not reached by most of our chosen β-nitrostyrene derivatives, due to the rather small core structure of β-nitrostyrene. Furthermore, TPSA-values indicated a good brain barrier permeability for all compounds (Pajouhesh and Lenz 2005). Overall indicating a humogen compound group. However, some of identified derivatives had LogP-values above three, whereas the LogP-values of test structures were found to be between 1.1878 (No. 5) and 2.3296 (No. 4). Therefore, in terms of LogP, the selected test-compounds have a reduced descriptor space.

As previously elaborated (Arif et al. 2009; Nicola et al. 2015; Franco et al. 2017; Boldini et al. 2024), a comprehensive subset of molecular fingerprints was used to compute similarity values. These can be categorized into structural key (PubChem, MACCS, EState) and topological descriptors. The latter can be subdivided into path- (AtomPair, Torsion, Avalon), daylight- (CDK Standard, CDK Extended, RDKit, Layerd) and circular-based (Morgan, FeatMorgan and CDK Circular) fingerprints. Despite being less complex, the structural key PubChem fingerprint was used as a reference, because it generated a broad spectrum of similarity values, showing its applicability for this dataset. As expected, related fingerprints, such as CDK Standard and CDK Extended, generated similar values. In contrary, the closely related fingerprints RDKit Morgan and RDKit FeatMorgan differed in the computed values, however, both followed the same pattern. Due to their multifactorial nature, computed similarity values have to be reviewed in a case by case manner (Maggiora et al. 2014). Further, a high structural similarity does not automatically imply biologic similarity. This can exemplary be highlighted by compound no. 2, being computed to be highly similar to β-nitrostyrene by the CDK PubChem and RDKit Pattern, with values of 0.877 and 0.907, respectively, whilst having no similarity of biological effects, as demonstrated in this study. In consequence, a clear similarity threshold for deriving similar biological effects can not be given, based on the geno(toxicty) results obtained in this study.

Among others, Ames test-based mutagenicity QSAR-alerts influenced the selection process. However, given that the core structure possesses antibiotic properties (Milhazes et al. 2006), which may explain the conflicting prediction results, a thorough review of bacteria-based test alerts is indicated. To clarify support these predictions and to estimate mutagenicity in mammalian cells, a mouse lymphoma assay was performed (OECD 2016b). As a result, a biologically relevant mutagenic potential was observed for β-nitrostyrene at 5 µM, confirming the positive QSAR alert for compound no. 1.

The QSAR mutagenicity alerts for the nine test compounds largely align with the literature and the test results in this study. However, the QSAR algorithms struggle to accurately predict pro-mutagens. Furthermore, compound no. 4 was identified as mutagenic by all algorithms, despite being a pro-mutagen (Bond 1989). Similarly, compound no. 6 was predicted to be non-mutagenic, but obviously exhibits pro-mutagenic properties (Hsu et al. 2007). This highlights the necessity for a thorough review of QSAR predictions, due to the risk of false negatives and false positives alerts.

The reason why no compounds was included to investigate the phenyl group of β-nitrostyrene can partly be given by the similarity comparisons against β-nitrostyrene, which favored compounds possessing a phenyl moiety. Additionally, Doré and Viel reported that the phenyl group seems important, but not essential for the cytotoxic effects of β-nitrostyrene, as it can be replaced by e.g., multiple conjugated double bonds or polycyclic structures without impairing its cytotoxicity (Doré and Viel 1975). The latter was also demonstrated in vitro in a more recent publication (Byrne et al. 2023).

In conclusion, a category approach was used to form a compound group, which adhered to the Lipinski rule of five. Structures lacking nitrovinyl groups were included for validity reasons, which however broadens the similarity values range. Nonetheless, a common functional group is not a prerequisite for compounds to be grouped together, as discussed in Sündermann et al. (2024).

### Cytotoxicity and genotoxicity

Clastogenicity, a predominant mode of action of, e.g., anti-neoplastic drugs, was evaluated in two different cell lines, i.e., L5178Y/TK^±^ mouse lymphoma cells and normal human WS1 skin fibroblasts from a black donor. L5178Y/TK^±^ cells are a standard cell line in genotoxicity testing and are a model system for mutagenicity testing according to OECD No. 490. WS1 cells were included in the study as primary human cell type and due to the unexpected pan-staining pattern of γH2A.X in mouse lymphoma cells, which is not ideal for quantification of DNA damage. This is mostly attributed to the fact that a pan-stained γH2A.X phenotype can also be associated with cellular senescence (Heckenbach et al. 2022) or apoptosis (Gasparri et al. 2004), endpoints not chosen for our investigation. As the staining intensity classifier used in this study is not able to differentiate between pan-stained cells and non-pan-stained cells, we aimed for a cell model possessing a low background of pan-staining, which was given by WS1 cells.

The observed difference in the γH2A.X phenotype might be linked to the p53-status of the cell lines. Opposing to the normal human WS1 skin fibroblast, L5178Y/TK^±^ cells express nonfunctional p53 (Storer et al. 1997). This might explain the pan-staining of these cells as p53 amongst other functions mediates the dephosphorilation of γH2A.X via wild-type p53-induced phosphatase 1 (WIP1, alias PPM1D), thus resulting in the elevated γH2A.X staining intensity observed in L5178Y/TK^±^ cells (Macůrek et al. 2010; Moon et al. 2010), due to inhibited dephosphorylation. Furthermore, a persisting DNA-damage response might lead to cell cycle arrest and senescence via the ubiquitin degradation of cell division cycle 25 homolog A (CDC25A), even if wildtype p53 is lacking (Mailand et al. 2000).

The performed alkaline comet assay experiment revealed that compounds comprising a nitrovinyl moiety exhibited a DNA damaging potential in L5178Y/TK^±^ cells. These compounds, except compound no. 9, were able to induce DNA double strand brakes in WS1 cells, as indicated by an increase in γH2A.X foci. This suggests that compound no. 9 only induces DNA single-strand breaks. The literature regarding in vitro clastogenicity of β-nitrostyrene and its derivatives is limited. There are only published data in scientific literature for a compound named 2-methoxy-5-[(E)-2-nitroprop-1-enyl]phenol (CYT-RX20), which is comparable to compound no. 9, but with an additional methyl-group in β-position (Tsai et al. 2017b; Wang et al. 2017a, b). These publications show the induction of DNA-double strand breaks by CYT-RX20. This highlights that even minor alterations to the core structure can significantly impact adverse biological reactivity.

The in vivo alkaline comet assay OECD guideline, i.e., No. 489 recommends the use of the median for slide summarizing measure (OECD 2016a), but in this study, we used the arithmetic mean. This was in spite the fact that the median is considered to be more robust in the case of skewed data distributions, which is often the case for comet assay data. However, the median is likely to underestimate the DNA-damaging effects, when induction of DNA damage is not present in more than 50% of the analyzed cells per slide. Thus, to account for the bi-modal distribution in TI, induced by several nitrovinyl moiety comprising compounds, as seen in Fig. S11, the arithmetic mean was finally chosen as summarizing measure. The finding that the DNA of a small fraction of cells was highly damaged, whereas a higher fraction of nuclei remained undamaged, correlated well with the hypothesis that derivatives with nitrovinyl moiety primarily induce DNA damage in dividing cells by acting as a topoisomerase II poison (Ziemann et al. 2006). Thus, the DNA-damaging effect seems to be limited to cells undergoing mitosis.

The known cytotoxic properties of β-nitrostyrene and its derivatives was assessed using two endpoints, i.e., LDH release as a marker for membrane permeability and turnover of WST-1, as measure for metabolic activity of the cell. While results for these endpoints exhibited a similar trend, the extent of reported cytotoxicity varied significantly between the two cell lines. This aligns with reports that the kind and origin of cell lines/cells can have a great influence on cytotoxicity readouts (Malich et al. 1997).

In line with the present study, β-nitrostyrene (compound no. 1) induced cytotoxic effects in vitro in a concentration range of 5 to 50 µM in previous studies (Doré and Viel 1975; Kaap et al. 2003; Mohan et al. 2006; Werner et al. 2007). For compound no. 3 the effective concentration range was reported to be 5 to 25 µM (Doré and Viel 1975; Kaap et al. 2003; Mohan et al. 2006). This aligns with our findings, as compound no. 3 was more cytotoxic than β-nitrostyrene. The cytotoxic potential of compounds no. 8 and 9 was also in line with literature reports (Mohan et al. 2006; Hsieh et al. 2010), as the reported effects were below the upper threshold reported for the lead structure. Furthermore, derivatives lacking a nitrovinyl moiety, in the present study, did not induce neither cytotoxic nor clastogenic effects in both WS1 and L5178Y/TK^±^, which was in line with literature reports (Doré and Viel 1975; Bond 1989; Kaap et al. 2003; Chung et al. 2006; Hsu et al. 2007; Taner et al. 2017; Salillas et al. 2019; Ugheighele et al. 2020). These reports were mainly based on LC_50_-values, published for rat pituitary GH3 tumor cells (Kaap et al. 2003), HeLa cells (Mohan et al. 2006; Salillas et al. 2019) and LoVo cells (Werner et al. 2007) as well as HBL cells (Hsieh et al. 2010). In alignment with this study (Ugheighele et al. 2020) used a fibroblasts cell line, namely 3T3 cells, which are of rodent origine. Qualitative cytotoxicity measures were also given for Leukemia L1210 cells (Doré and Viel 1975). In the majority of these publications, metabolic status of the cells served as main endpoint by using XTT (Salillas et al. 2019) and MTT assays (Werner et al. 2007; Hsieh et al. 2010; Ugheighele et al. 2020), whereas the WST-1 assay was used in the present study. Additionally, protein content by an sulforhodamine B assay (Mohan et al. 2006) or a live/dead staining using methylene (Kaap et al. 2003) served as methods to estimate cytotoxicity of β-nitrostyrene derivatives.

In vivo studies have demonstrated relevant cytotoxic effects for the lead structure β-nitrostyrene (Doré and Viel 1975) which, in the case of leukemia 1210 ascites cancer, were even detectable at concentrations without anti-neoplastic activity. Tsai et al. (2017a, b) have tested the β-nitrostyrene derivative CYT-RX20, dosed in a volume of 100 µL with a concentration of 35.8 µM in vivo, demonstrated a significant reduction of tumor weight after three weeks of treatment with 3 doses per week in female immune-deficient BALB/cAnN.Cg-Foxn1nu/CrlNarl mice xenograft model using human HCT116 colon cancer cells (Tsai et al. 2017b). Nonetheless, elevated GOT and GPT levels point towards liver toxicity of this β-nitrostyrene derivative.

To test for aneugenicity, the mitotic marker pH3 (S10) was selected in the present study. As previously reported for three human cancer cell lines, namely HepG2, LS-174T and ACHN (Khoury et al. 2016), either an increase or a decrease in the phosphorylation of histone 3 is expected for aneugens, depending on their aneugenic mode of action. In this study, neither an increase nor decrease in phosphorylation of histone 3 was observed, suggesting that all test compounds lacked aneugenic potential. However, since some test compounds in this study were markedly clastogenic, a reduction in the phosphorylation of pH3 (S10) was expected, as DNA-damage inhibits the kinase responsible for the phosphorylation of histone 3 (Monaco et al. 2005). Nonetheless, Khoury et al. (2016) reported that known clastogens, such as benzo[a]pyrene and MMS did not alter pH3 (S10) frequency, which is in line with our results. Furthermore, the characteristic profile for clastogens was an induction of γH2A.X at lower and decrease of pH3 (S10) at high concentrations. For instance, Etoposide induced an significant increase in γH2A.X in HepG2 cells at 0.1 µM, whilst a significant reduction in pH3 (S10) was reported at 10 µM (Khoury et al. 2016). Thus, it might be necessary to increase the test compound concentration to certainly measure an alteration in histone 3 phosphorylation. However, higher test compound concentrations might induce non-specific cytotoxicity-induced genotoxic effects.

### Conclusion

In conclusion, considering the present results for the β-nitrostyrene derivatives without nitrovinyl moiety (compounds no. 2, 4, 6, and 7), the nitrovinyl group seems key for the cytotoxic and genotoxic potential of β-nitrostyrene, at least in L5178Y/TK^±^ and WS1 cells, as alteration or substitution of this group changed or even negated these adverse biological effects. An additional nitro group in ortho position (compound no. 3) increases the cytotoxic and clastogenic potential compared to the lead-structure β-nitrostyrene, as did the dioxol group (compound no. 8), which markedly potentiated the adverse biological activity. In addition, hydroxy and methoxy groups in meta and para position to the nitrovinyl group (compound no. 9), can enhance these effects, although its ability to induce double stand breaks seemed to be hindered.

The goal of identifying the structural elements responsible for the adverse biological effects of β-nitrostyrene was achieved. The knowledge, that the nitrovinyl moiety is the main driver for the (geno)toxic properties of β-nitrostyrene and its derivatives, and the understanding that these effects can be modulated by functionalization can now be leveraged for the development of safer substances wherever needed.

## Supplementary Information

Below is the link to the electronic supplementary material.

Supplementary Material 1

## Data Availability

The datasets generated during this study are available from corresponding author on request.

## Abbreviations

ANOVA: Analysis of variance
BSA: Bovine serum albumin
CDK: Chemistry Development Kit
CE: Cloning efficiency
CAS: Chemical Abstracts Service
DAPI: 4′,6-Diamidin-2-phenylindol
DMSO: Dimethyl sulfoxide
ECHA: European Chemicals Agency
EFSA: European Food Safety Authority
HPVC: United States High Production Volume Challenge Program List
ICH: International Council for Harmonisation of Technical Requirements for Pharmaceuticals for Human Use
IUPAC: International Union of Pure and Applied Chemistry
KNIME: Konstanz Information Miner
LDH: Lactate dehydrogenase
MF: Mutation frequency
MLA: Mouse lymphoma assay
MMS: Methyl methanesulfonate
NMA: Normal melting agarose
OECD: Organisation for Economic Co-operation and Development
pH3 (S10): Phosphorylated serin 10 on Histon H3
QSAR: Quantitative structure–activity relationship
RCE: Relative cloning efficiency
REACh: Registration, Evaluation, Authorisation and Restriction of Chemicals
RSG: Relative suspension growth
SD: Standard deviation
SG: Suspension growth
SMILES: Simplified molecular-input line-entry system
TBS: Tris-buffered saline
TI: Tail intensity
WST-1: Water soluble tetrazolium salt
γH2A.X: Histone H2A.X phosphorylated on serin 139

## Footnotes

1 https://cdxapps.epa.gov/oms-substance-registry-services/substance-list-details/74—10.10.2024.
