# Supplementary material for: The nitrovinyl moiety determines the cyto- and genotoxic profiles of β-nitrostyrene derivatives: evidence from in silico and in vitro evaluation
Source: Arch Toxicol. 2026 Mar 31;100(6):2647–64. doi: 10.1007/s00204-026-04349-4 (PMC13221336; doi:10.1007/s00204-026-04349-4)
Supplement: Supplementary file 1 — Supplementary Material 1 [file 204_2026_4349_MOESM1_ESM.docx]

# Supplementary Material

## pH3 (S10) Classifier Settings:

*Capture:* The DAPI Channel (Channel Name) with an automatic “Exposure Mode” was selected as counterstaining. The maximum integration time ([Max.] T Integ.) was set to 0.08 s as well as the saturation area to 400 (1/100 µm^2^). “No. Of Fucus Planes” was set to 1 and the “Foc. Pl. Distance” to 0 (1/40 µm). “Signal 1” (pH3 S[S10]) was captured in the red Cy5 Chanel (Chanel Name) with an automatic “Exposure Mode”. The integration Time ([Max.] T Integ.) was set to 0.12 s with the Saturation Area adjusted to 100 (1/100 µm^2^). “No. Of Focus Planes” was set to 5 and the “Foc. Pl. Distance” to 40 (1/10 µm). Further, the 40X lens was selected (Microscope Magnification) and “CCD Camera Gain” was increased to 5.0. The DAPI Chanel was used as a mask for the cell nuclei detection (Use Counterstain Mask for Capturing).

*Exposure:* “No. of Exposures” was set to 1, “Integ. Time Factor” to 5 and “Min. T Integ” to 0 s for both channels. “Initial T Integration” was adjusted to 25 %, „Fixed Lamp Intensity” to 0 and Lamp RGB Levels were set to 1000 each.

*Cell Selection (Single Cells):* “Minimum Object Area” was adjusted to 50 µm^2^ and the “Maximum Object Area” to 250 µm^2^. “Max. Concavity Depth” was set to 0.25 and “Maximum Aspect Ratio” to 2.5. The “CS Object Threshold” was adjusted to 20 %.

*Cell Proc.:* For both channels “SBHistoMax ApplyMask” at “Operations for Cell Image/Analysis” was selected and “Extend Counterstain Mask” was set to 5 /10 µm.

*Features:* The features for final classification were listed in this menu. For the DAPI channel following feature was assessed:

1. Total Area within Contour in µm^2^

For the Cy5 channel (pH3 [S10]) the following features were utilized for the assessment:

1. Integrated Intensity within Contour
2. Total Relative Area at 20 % Intensity (Maximum Gain 100%, Upper Thr. With 2 Sat. Pixels)
3. Ratio Value of Feature Variables 3 and 2 (Scale Factor 100=1.0)
4. Box Classifier (“4(0.5,)”)
5. Box Classifier (“2(400000,)”)
6. Logic Operation 2 between Feature 5 and Feature 6

Further, “Max. Spot Area” was set to 100 and “Min. Spot Contrast” to 200.

*Gallery:* Gallery Images were activated and the “Gallery Image Size” was adjusted to 4. “Gallery Scale” was set to 100 % with no “Gallery Compression”. As “Color Mode” the “RGB Composite” was chosen. The SC channel (DAPI) was displayed in blue, while the Cy5 channel was displayed in red. “Lower Thr. Algorithm die Min. Intensity” and “Upper Thr. Algorithm” (Max. Intensity) were selected for post processing within the DAPI channel. “*Lower Thr. Offset*” was set to 0 for both channels. The “UT Sat. Area” and the “Maximum Gain” was set to 10 (1/100 µm^2^) and to 200% for both channels. The “Rel. Intensity” for the DAPI channel was set to 50 % and for the Cy5 channel to 100%.

*Other:* “Field of View Images” and “Trainings Images” were selected.

### γH2A.X Classifier:

The following parameters were different from the pH3(S10) Classifier:

*Capture:* Signal 1 (γH2A.X) was captures in the green Cy3 channel (Channel Name). The instigation time ([Max.] T Integ.) was set to 1.04 s with the “Saturation Area” adjusted to 100 (1/100 µm^2^).

*Features:* The features for final classification of the Cy3 channel are listed below.

1. *Integrated Intensity within Contour*
2. *Box Classifier (“2(8000,)”)*

*Gallery:* For the Cy3 channel a green “Display Color” was chosen.

**Tab. S1:** **Dataset based on literature review and database search.**

| ID. | No. | IUPAC Name | CAS-Nr. | SMILES | Structure |
| --- | --- | --- | --- | --- | --- |
| 1 | 1 | [(E)-2-nitroethenyl]  benzene | 102-96-5 | C1=CC=C(C=C1)C=C  [N+](=O)[O-] |  |
| 2 | 2 | (2-Nitroethyl)  benzene | 6125-24-2 | C1=CC=C(C=C1)CC  [N+](=O)[O-] |  |
| 3 | - | 1-chloro-4-[(E)-  2-nitroethenyl]  benzene | 706-07-0 | C1=CC(=CC=C1C=C  [N+](=O)[O-])Cl |  |
| 4 | - | (E)-2-phenyl  ethenamine | 5694-20-2 | C1=CC=C(C=C1)C=CN |  |
| 5 | 3 | 1-nitro-2[(E)-2-  itroethenyl]benzene | 3156-39-6 | C1=CC=C(C(=C1)C=C[N+]  =O)[O-])[N+](=O)[O-] |  |
| 6 | 4 | Styrene | 100-42-5 | C=CC1=CC=CC=C1 |  |
| 7 | 5 | 2-phenylethanamine | 64-04-0 | C1=CC=C(C=C1)CCN |  |
| 8 | - | 2-[(E)-2-nitroethenyl]  phenol | 3156-43-2 | C1=CC=C(C(=C1)C=C  [N+](=O)[O-])O |  |
| 9 | 6 | Nitrobenzene | 98-95-3 | C1=CC=C(C=C1)  [N+](=O)[O-] |  |
| 10 | - | 1-methoxy-2-[(E)  -2-nitroethenyl]  benzene | 3316-24-3 | COC1=CC=CC=C1C=C  [N+](=O)[O-] |  |
| 11 | 7 | (E)-3-phenylprop-  2-enoic acid | 140-10-3 | C1=CC=C(C=C1)  C=CC(=O)O |  |
| 12 | - | 1,4-dimethoxy-2-  [(E)-2-nitroethenyl]  benzene | 40276-11-7 | COC1=CC(=C(C=C1)OC)  C=C[N+](=O)[O-] |  |
| 13 | - | 1,2-dimethoxy-4-  [(E)-2-nitroethenyl]  benzene | 4230-93-7 | COC1=C(C=C(C=C1)C=C  [N+](=O)[O-])OC |  |
| 14 | - | 1,3,5-Trimethoxy-  2-(2-nitroethenyl)  benzene | 216433-58-8 | COC1=CC(=C(C(=C1)OC)  C=C[N+](=O)[O-])OC |  |
| 15 | - | 4-bromo-1-methoxy  -2-[(E)-2-nitroethen  yl]benzene | 175205-14-8 | COC1=C(C=C(C=C1)Br)  C=C[N+](=O)[O-] |  |
| 16 | 8 | 5-[(E)-2-nitroethen  yl]-1,3-benzo  dioxole | 1485-00-3 | C1OC2=C(O1)C=C(C=C2)  C=C[N+](=O)[O-] |  |
| 17 | - | 3-(4-methoxyphen  yl)-2-nitroprop-2  -en-1-ol | 905564-22-9 | COC1=CC=C(C=C1)  C=C(CO)[N+](=O)[O-] |  |
| 18 | 9 | 2-methoxy-5-[(E)  -2-nitroethenyl]  phenol | 39816-35-8 | COC1=C(C=C(C=C1)  C=C[N+](=O)[O-])O |  |
| 19 | - | 2-methoxy-4-  [(E)-2-nitroethenyl]  phenol | 6178-42-3 /  22568-51-0 | COC1=C(C=CC(=C1)  C=C[N+](=O)[O-])O |  |
| 20 | - | 1,2,3-Trimethoxy-5-  [(E)-2-nitrovinyl]  benzene | 6316-70-7 | COC1=CC(=CC(=C1OC)  OC)C=C[N+](=O)[O-] |  |
| 21 | - | 2-ethoxy-4-  [(e)-2-nitroethenyl]  phenol | 90922-85-3 | CCOC1=C(C=CC(=C1)  C=C[N+](=O)[O-])O |  |
| 22 | - | 4-Bromo-1-methoxy-  2-(2-nitrobut-1-enyl)  benzene | AKOS /  17433849 | CCC(=CC1=C(C=CC(=C1)  Br)OC)[N+](=O)[O-] |  |
| 23 | - | 3-(3,4-dimethoxyphenyl)-  2-nitroprop-2-en-1-ol | 915161-59-0 | COC1=C(C=C(C=C1)C=C  (CO)[N+](=O)[O-])OC |  |
| 24 | - | 2-Ethoxy-4-[(E)-2-  nitroprop-1-enyl]  phenol | AC1LF7G6 | CCOC1=C(C=CC(=C1)C=C  (C)[N+](=O)[O-])O | 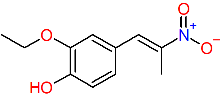 |
| 25 | - | 2-nitro-3-thiophen-2-  ylprop-2-en-1-ol | 915161-55-6 | C1=CSC(=C1)C=C(CO)  [N+](=O)[O-] |  |
| 26 | - | 3-[(E)-2-nitroethenyl]-  1H-indole | 3156-51-2 | C1=CC=C2C(=C1)C(=CN2)  C=C[N+](=O)[O-] |  |
| 27 | - | 5-[(Z)-3-(1,3-benzodioxol-5-yl)-2-nitroprop-2-enyl]-1,3-benzodioxole | CHEMBL / 1672014 | C1OC2=C(O1)C=C(C=C2)  CC(=CC3=CC4=C(C=C3)  OCO4)[N+](=O)[O-] |  |
| 28 | - | (2R)-7-methoxy-  3-nitro-2-phenyl-  2H-chromene | CTK8G5115 | COC1=CC2=C(C=C1)  C=C(C(O2)C3=CC=CC=C3)  [N+](=O)[O-] |  |
| 29 | - | (E)-3-(2-Nitrovinyl)  furan | 122016-40-4 | C1=COC=C1C=C  [N+](=O)[O-] |  |


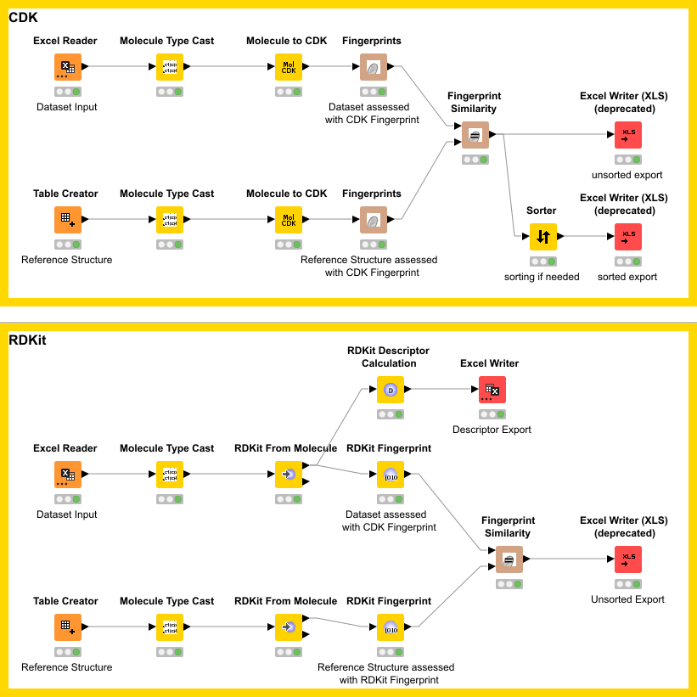


**Fig. S1:** **KNIME workflow to compute fingerprint based chemical similarity values as well as chemical properties.** The upper yellow bow harbors the CDK fingerprint nodes whereas the lower boy contains the RDKit fingerprint and descriptor nodes.

**Tab. S2:** **Values of the Tanimoto values based on different fingerprints.** The first column indicates the compound number within the entire dataset. The second column indexes the test-compounds.

| IDccc | No. | CDK-PubChem | CDK-MACCS | CDK-Circular | CDK-Estate | CDK-Standard | CDK-Extended | RDKit-Pattern | RDKit-Layered | RDKit-MACCS | RDKit-Avalon | RDKit-FeatMorgan | RDKit-AtomPair | RDKit-RDKit | RDKit-Morgan | RDKit-Torsion |
| --- | --- | --- | --- | --- | --- | --- | --- | --- | --- | --- | --- | --- | --- | --- | --- | --- |
| 1 | 1 | 1.000 | 1.000 | 1.000 | 1.000 | 1.000 | 1.000 | 1.000 | 1.000 | 1.000 | 1.000 | 1.000 | 1.000 | 1.000 | 1.000 | 1.000 |
| 2 | 2 | 0.877 | 0.657 | 0.714 | 0.667 | 0.338 | 0.362 | 0.907 | 0.719 | 0.690 | 0.471 | 0.478 | 0.593 | 0.281 | 0.355 | 0.353 |
| 3 | - | 0.859 | 0.867 | 0.857 | 0.833 | 0.804 | 0.824 | 0.832 | 0.848 | 0.846 | 0.800 | 0.727 | 0.623 | 0.770 | 0.630 | 0.400 |
| 4 | - | 0.831 | 0.464 | 0.429 | 0.500 | 0.664 | 0.675 | 0.702 | 0.723 | 0.292 | 0.408 | 0.364 | 0.360 | 0.638 | 0.423 | 0.636 |
| 5 | 3 | 0.770 | 0.813 | 1.000 | 1.000 | 0.698 | 0.712 | 0.685 | 0.593 | 0.710 | 0.643 | 0.696 | 0.553 | 0.394 | 0.586 | 0.381 |
| 6 | 4 | 0.750 | 0.250 | 0.429 | 0.500 | 0.619 | 0.619 | 0.629 | 0.554 | 0.174 | 0.357 | 0.421 | 0.300 | 0.480 | 0.346 | 0.583 |
| 7 | 5 | 0.737 | 0.263 | 0.250 | 0.286 | 0.314 | 0.327 | 0.646 | 0.517 | 0.156 | 0.208 | 0.250 | 0.271 | 0.267 | 0.194 | 0.400 |
| 8 | - | 0.713 | 0.765 | 0.857 | 0.833 | 0.729 | 0.770 | 0.780 | 0.723 | 0.710 | 0.655 | 0.696 | 0.661 | 0.623 | 0.552 | 0.471 |
| 9 | 6 | 0.675 | 0.821 | 0.833 | 0.800 | 0.220 | 0.250 | 0.759 | 0.507 | 0.800 | 0.468 | 0.318 | 0.400 | 0.161 | 0.423 | 0.375 |
| 10 | - | 0.670 | 0.722 | 0.750 | 0.714 | 0.651 | 0.693 | 0.747 | 0.636 | 0.667 | 0.720 | 0.667 | 0.588 | 0.492 | 0.485 | 0.421 |
| 11 | 7 | 0.639 | 0.290 | 0.500 | 0.571 | 0.457 | 0.459 | 0.729 | 0.675 | 0.231 | 0.477 | 0.346 | 0.623 | 0.359 | 0.429 | 0.538 |
| 12 | - | 0.626 | 0.684 | 0.750 | 0.714 | 0.557 | 0.598 | 0.653 | 0.557 | 0.629 | 0.643 | 0.538 | 0.361 | 0.403 | 0.368 | 0.192 |
| 13 | - | 0.615 | 0.667 | 0.750 | 0.714 | 0.548 | 0.563 | 0.663 | 0.583 | 0.611 | 0.643 | 0.667 | 0.333 | 0.443 | 0.471 | 0.240 |
| 14 | - | 0.604 | 0.650 | 0.750 | 0.714 | 0.494 | 0.527 | 0.569 | 0.526 | 0.595 | 0.618 | 0.560 | 0.250 | 0.388 | 0.361 | 0.094 |
| 15 | - | 0.593 | 0.650 | 0.667 | 0.625 | 0.584 | 0.615 | 0.649 | 0.526 | 0.595 | 0.581 | 0.467 | 0.424 | 0.381 | 0.359 | 0.200 |
| 16 | 8 | 0.588 | 0.565 | 0.750 | 0.714 | 0.462 | 0.473 | 0.599 | 0.526 | 0.550 | 0.318 | 0.640 | 0.386 | 0.396 | 0.457 | 0.222 |
| 17 | - | 0.587 | 0.565 | 0.545 | 0.500 | 0.445 | 0.475 | 0.639 | 0.577 | 0.524 | 0.493 | 0.387 | 0.337 | 0.443 | 0.238 | 0.160 |
| 18 | 9 | 0.578 | 0.667 | 0.667 | 0.625 | 0.523 | 0.547 | 0.689 | 0.593 | 0.611 | 0.554 | 0.571 | 0.409 | 0.460 | 0.432 | 0.261 |
| 19 | - | 0.578 | 0.667 | 0.667 | 0.625 | 0.541 | 0.563 | 0.689 | 0.599 | 0.611 | 0.563 | 0.571 | 0.389 | 0.483 | 0.432 | 0.261 |
| 20 | - | 0.573 | 0.634 | 0.750 | 0.714 | 0.460 | 0.457 | 0.569 | 0.526 | 0.579 | 0.586 | 0.625 | 0.236 | 0.398 | 0.441 | 0.172 |
| 21 | - | 0.558 | 0.591 | 0.600 | 0.556 | 0.460 | 0.469 | 0.663 | 0.569 | 0.550 | 0.529 | 0.552 | 0.340 | 0.414 | 0.400 | 0.250 |
| 22 | - | 0.552 | 0.591 | 0.545 | 0.500 | 0.397 | 0.411 | 0.541 | 0.441 | 0.512 | 0.413 | 0.270 | 0.288 | 0.286 | 0.188 | 0.063 |
| 23 | - | 0.542 | 0.542 | 0.545 | 0.500 | 0.383 | 0.394 | 0.549 | 0.479 | 0.478 | 0.418 | 0.375 | 0.227 | 0.339 | 0.222 | 0.091 |
| 24 | - | 0.520 | 0.578 | 0.545 | 0.500 | 0.368 | 0.378 | 0.574 | 0.507 | 0.512 | 0.418 | 0.364 | 0.264 | 0.356 | 0.213 | 0.065 |
| 25 | - | 0.515 | 0.556 | 0.600 | 0.556 | 0.298 | 0.326 | 0.595 | 0.384 | 0.525 | 0.207 | 0.265 | 0.400 | 0.235 | 0.244 | 0.174 |
| 26 | - | 0.493 | 0.703 | 0.857 | 0.714 | 0.427 | 0.450 | 0.561 | 0.533 | 0.667 | 0.193 | 0.517 | 0.456 | 0.304 | 0.457 | 0.280 |
| 27 | - | 0.492 | 0.531 | 0.600 | 0.625 | 0.272 | 0.276 | 0.376 | 0.436 | 0.468 | 0.256 | 0.375 | 0.189 | 0.252 | 0.208 | 0.061 |
| 28 | - | 0.489 | 0.542 | 0.500 | 0.556 | 0.241 | 0.238 | 0.373 | 0.331 | 0.468 | 0.212 | 0.353 | 0.266 | 0.182 | 0.216 | 0.163 |
| 29 | - | 0.432 | 0.806 | 0.857 | 0.833 | 0.368 | 0.406 | 0.755 | 0.592 | 0.778 | 0.344 | 0.560 | 0.660 | 0.441 | 0.533 | 0.400 |

**Fig. S2: Heatmap visualization of fingerprint similarity of entire dataset.**

**Fig. S3:** **Chemical descriptors computed for the entire dataset.**

**Tab. S 3: Chemical descriptors calculated with RDKit utilizing KNIME.**

| ID | No. |  | SlogP | TPSA | AMW | Lipinski HBA | Lipinski HBD | Heavy Atoms | Rotatable Bonds |
| --- | --- | --- | --- | --- | --- | --- | --- | --- | --- |
| 1 | 1 |  | 1.934 | 43.14 | 149.149 | 3 | 0 | 11 | 2 |
| 2 | 2 |  | 1.5058 | 43.14 | 151.165 | 3 | 0 | 11 | 3 |
| 3 | - |  | 2.5874 | 43.14 | 183.594 | 3 | 0 | 12 | 2 |
| 4 | - |  | 1.616 | 26.02 | 119.167 | 1 | 2 | 9 | 1 |
| 5 | 3 |  | 1.8422 | 86.28 | 194.146 | 6 | 0 | 14 | 3 |
| 6 | 4 |  | 2.3296 | 0 | 104.152 | 0 | 0 | 8 | 1 |
| 7 | 5 |  | 1.1878 | 26.02 | 121.183 | 1 | 2 | 9 | 2 |
| 8 | - |  | 1.6396 | 63.37 | 165.148 | 4 | 1 | 12 | 2 |
| 9 | 6 |  | 1.5948 | 43.14 | 123.111 | 3 | 0 | 9 | 1 |
| 10 | - |  | 1.9426 | 52.37 | 179.175 | 4 | 0 | 13 | 3 |
| 11 | 7 |  | 1.7844 | 37.3 | 148.161 | 2 | 1 | 11 | 2 |
| 12 | - |  | 1.9512 | 61.6 | 209.201 | 5 | 0 | 15 | 4 |
| 13 | - |  | 1.9512 | 61.6 | 209.201 | 5 | 0 | 15 | 4 |
| 14 | - |  | 1.9598 | 70.83 | 239.227 | 6 | 0 | 17 | 5 |
| 15 | - |  | 2.7051 | 52.37 | 258.071 | 4 | 0 | 14 | 3 |
| 16 | 8 |  | 1.6627 | 61.6 | 193.158 | 5 | 0 | 14 | 2 |
| 17 | - |  | 1.3051 | 72.6 | 209.201 | 5 | 1 | 15 | 4 |
| 18 | 9 |  | 1.6482 | 72.6 | 195.174 | 5 | 1 | 14 | 3 |
| 19 | - |  | 1.6482 | 72.6 | 195.174 | 5 | 1 | 14 | 3 |
| 20 | - |  | 1.9598 | 70.83 | 239.227 | 6 | 0 | 17 | 5 |
| 21 | - |  | 2.0383 | 72.6 | 209.201 | 5 | 1 | 15 | 4 |
| 22 | - |  | 3.4853 | 52.37 | 286.125 | 4 | 0 | 16 | 4 |
| 23 | - |  | 1.3137 | 81.83 | 239.227 | 6 | 1 | 17 | 5 |
| 24 | - |  | 2.4284 | 72.6 | 223.228 | 5 | 1 | 16 | 4 |
| 25 | - |  | 1.358 | 63.37 | 185.204 | 4 | 1 | 12 | 3 |
| 26 | - |  | 2.4153 | 58.93 | 188.186 | 4 | 1 | 14 | 2 |
| 27 | - |  | 3.0043 | 80.06 | 327.292 | 7 | 0 | 24 | 4 |
| 28 | - |  | 3.4465 | 61.6 | 283.283 | 5 | 0 | 21 | 3 |
| 29 | - |  | 1.527 | 56.28 | 139.11 | 4 | 0 | 10 | 2 |

**Tab. S4: Ames-Test mutagenicity alerts by GT1_BMUT.**

| ID | No. | Positive  Feature | Deactivating  Feature | Unknown  Feature | Known  Structure | Prediction | Probability |
| --- | --- | --- | --- | --- | --- | --- | --- |
| 1 | 1 | 2 | 0 | 0 | **Yes** | **Known Negative** | 48,1 |
| 2 | 2 | 1 | 0 | 0 | No | **Inconclusive** | 45,8 |
| 3 | - | 2 | 0 | 0 | **Yes** | **Known Positive** | 48,1 |
| 4 | - | 1 | 0 | 1 | No | **Out of Domain** | 24,6 |
| 5 | 3 | 3 | 2 | 0 | No | **Inconclusive** | 56,3 |
| 6 | 4 | 0 | 0 | 0 | **Yes** | **Known Positive** | 22,9 |
| 7 | 5 | 0 | 0 | 0 | No | **Negative** | 22,9 |
| 8 | - | 2 | 0 | 0 | No | **Inconclusive** | 48,1 |
| 9 | 6 | 4 | 0 | 0 | **Yes** | **Known Negative** | 86,1 |
| 10 | - | 2 | 0 | 0 | No | **Inconclusive** | 48,1 |
| 11 | 7 | 1 | 0 | 0 | **Yes** | **Known Negative** | 24,6 |
| 12 | - | 3 | 0 | 0 | No | **Inconclusive** |  |
| 13 | - | 2 | 0 | 0 | No | **Inconclusive** | 48,1 |
| 14 | - | 6 | 0 | 0 | No | **Positive** | 82,7 |
| 15 | - | 2 | 1 | 0 | No | **Negative** | 38,8 |
| 16 | 8 | 3 | 0 | 0 | No | **Positive** | 61,7 |
| 17 | - | 3 | 0 | 0 | No | **Positive** | 70,5 |
| 18 | 9 | 2 | 0 | 0 | No | **Inconclusive** | 48,1 |
| 19 | - | 2 | 2 | 0 | No | **Negative** | 19,5 |
| 20 | - | 2 | 2 | 0 | No | **Negative** |  |
| 21 | - | 2 | 2 | 0 | No | **Negative** | 19,5 |
| 22 | - | 1 | 2 | 0 | No | **Negative** | 28,1 |
| 23 | - | 2 | 0 | 0 | No | **Positive** | 61,9 |
| 24 | - | 1 | 1 | 1 | No | **Out of Domain** | 31,9 |
| 25 | - | 2 | 0 | 0 | No | **Positive** | 61,9 |
| 26 | - | 7 | 0 | 0 | No | **Positive** | 78,6 |
| 27 | - | 2 | 0 | 0 | No | **Inconclusive** | 59,5 |
| 28 | - | 3 | 0 | 1 | No | **Positive** | 61,3 |
| 29 | - | 2 | 1 | 0 | No | **Negative** | 30,4 |

**Tab. S5:** **Ames-Test mutagenicity alerts by PHARM_BMUT.**

| ID | No. | Positive  Feature | Deactivating  Feature | Unknown  Feature | Known  Structure | Prediction | Probability |
| --- | --- | --- | --- | --- | --- | --- | --- |
| 1 | 1 | 4 | 0 | 0 | No | **Positive** | 58,2 |
| 2 | 2 | 1 | 1 | 0 | No | **Negative** | 17,6 |
| 3 | - | 4 | 0 | 0 | **Yes** | **Known Positive** | 58,2 |
| 4 | - | 1 | 0 | 1 | No | **Out of Domain** | 22,3 |
| 5 | 3 | 6 | 0 | 0 | No | **Positive** | 98,4 |
| 6 | 4 | 1 | 0 | 0 | **Yes** | **Known Positive** | 23,3 |
| 7 | 5 | 1 | 0 | 0 | No | **Negative** | 8,3 |
| 8 | - | 5 | 0 | 0 | No | **Positive** | 70,6 |
| 9 | 6 | 0 | 0 | 0 | **Yes** | **Known Negative** | 86,5 |
| 10 | - | 5 | 0 | 0 | No | **Positive** | 70,6 |
| 11 | 7 | 1 | 1 | 0 | **Yes** | **Known Negative** | 13,5 |
| 12 | - | 5 | 0 | 0 | No | **Positive** | 68,9 |
| 13 | - | 4 | 1 | 0 | No | **Inconclusive** | 35,3 |
| 14 | - | 5 | 0 | 0 | No | **Positive** | 69,5 |
| 15 | - | 6 | 0 | 0 | No | **Inconclusive** | 34,9 |
| 16 | 8 | 5 | 1 | 0 | No | **Positive** | 47,5 |
| 17 | - | 2 | 0 | 1 | No | **Positive** | 78 |
| 18 | 9 | 4 | 2 | 0 | No | **Negative** | 13,7 |
| 19 | - | 4 | 0 | 0 | No | **Positive** | 58,2 |
| 20 | - | 4 | 9 | 0 | No | **Positive** | 58,2 |
| 21 | - | 4 | 2 | 1 | No | **Inconclusive** | 35,5 |
| 22 | - | 1 | 2 | 1 | No | **Inconclusive** | 36,1 |
| 23 | - | 2 | 1 | 1 | No | **Positive** | 66,5 |
| 24 | - | 1 | 2 | 1 | No | **Out of Domain** | 24,5 |
| 25 | - | 2 | 0 | 1 | No | **Positive** | 78 |
| 26 | - | 7 | 1 | 0 | No | **Positive** | 87,9 |
| 27 | - | 2 | 2 | 1 | No | **Positive** | 49,9 |
| 28 | - | 1 | 0 | 1 | No | **Positive** | 59,5 |
| 29 | - | 4 | 0 | 0 | No | **Positive** | 58,2 |

**Tab. S6: Ames-Test mutagenicity alerts by GT_EXPERT.**

| ID | No. | Positive  Feature | Deactivating  Feature | Unknown  Feature | Known  Structure | Prediction | Prediction |
| --- | --- | --- | --- | --- | --- | --- | --- |
| 1 | 1 | 0,0 | 0,0 | 0 | Yes | **Known Negative** | 0 |
| 2 | 2 | 0,0 | 0,0 | 0 | No | **Negative** | 0 |
| 3 | - | 0,0 | 0,0 | 0 | Yes | **Known Positive** | 0 |
| 4 | - | 0,0 | 0,0 | 1 | No | **Out of Domain** | 0 |
| 5 | 3 | 1,0 | 0,0 | 0 | No | **Positive** | 87,8 |
| 6 | 4 | 0,0 | 0,0 | 0 | Yes | **Known Positive** | 0 |
| 7 | 5 | 0,0 | 0,0 | 0 | No | **Negative** | 0 |
| 8 | - | 0,0 | 0,0 | 0 | No | **Negative** | 0 |
| 9 | 6 | 1,0 | 0,0 | 0 | Yes | **Known Negative** | 87,8 |
| 10 | - | 0,0 | 0,0 | 0 | No | **Negative** | 0 |
| 11 | 7 | 0,0 | 0,0 | 0 | Yes | **Known Negative** | 0 |
| 12 | - | 0,0 | 0,0 | 0 | No | **Negative** | 0 |
| 13 | - | 0,0 | 0,0 | 0 | No | **Negative** | 0 |
| 14 | - | 0,0 | 0,0 | 0 | No | **Negative** | 0 |
| 15 | - | 0,0 | 0,0 | 0 | No | **Negative** | 0 |
| 16 | 8 | 0,0 | 0,0 | 0 | No | **Negative** | 0 |
| 17 | - | 0,0 | 0,0 | 0 | No | **Negative** | 0 |
| 18 | 9 | 0,0 | 0,0 | 0 | No | **Negative** | 0 |
| 19 | - | 0,0 | 0,0 | 0 | No | **Negative** | 0 |
| 20 | - | 0,0 | 0,0 | 0 | No | **Negative** | 0 |
| 21 | - | 0,0 | 0,0 | 0 | No | **Negative** | 0 |
| 22 | - | 0,0 | 0,0 | 0 | No | **Negative** | 0 |
| 23 | - | 0,0 | 0,0 | 0 | No | **Negative** | 0 |
| 24 | - | 0,0 | 0,0 | 1 | No | **Out of Domain** | 0 |
| 25 | - | 0,0 | 0,0 | 0 | No | **Negative** | 0 |
| 26 | - | 0,0 | 0,0 | 0 | No | **Negative** | 0 |
| 27 | - | 0,0 | 0,0 | 0 | No | **Negative** | 0 |
| 28 | - | 0,0 | 0,0 | 1 | No | **Out of Domain** | 0 |
| 29 | - | 0,0 | 0,0 | 0 | No | **Negative** | 0 |


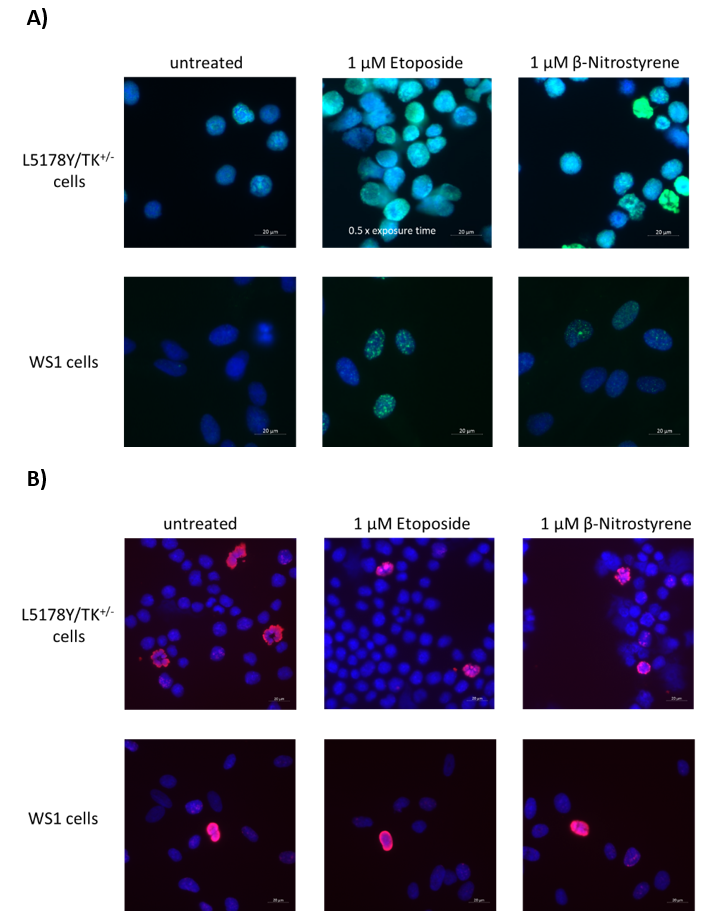


**Fig. S4: Representative** **γH2A.X und pH3 (S10) stained WS1 and L5178Y/TK^+/-^ cells.** The γH2A.X staining is seen in **A)** whereas **B)** shows the pH3 (S10) stained cells after an 4 h incubation (1 h for Etoposide).


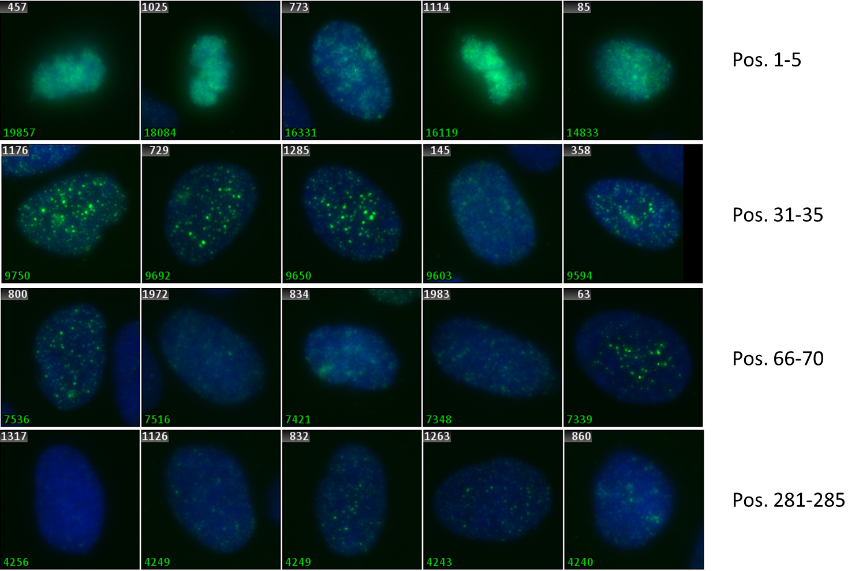


**Fig. S5:** **Representative γH2A.X stained WS1 cells sorted by fluorescence intensity.** Above the threshold of 8000 fluorescence units, which is indicated as a green number in the lower left corner of each picture, cells are classified positively.


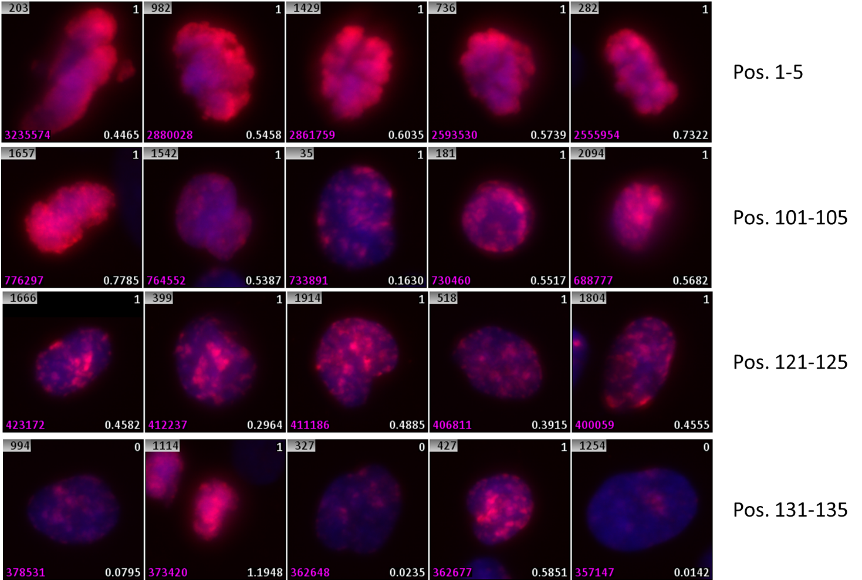


**Fig. S6:** **Representative pH3 (S10) stained WS1 cell nuclei, sorted by staining intensity.** A low staining intensity is mainly found in non-mitotic cells. However, cells during anna- and telophase also represent a low staining intensity. This is because their staining intensity is divided between two daughter cell nuclei (position 132).


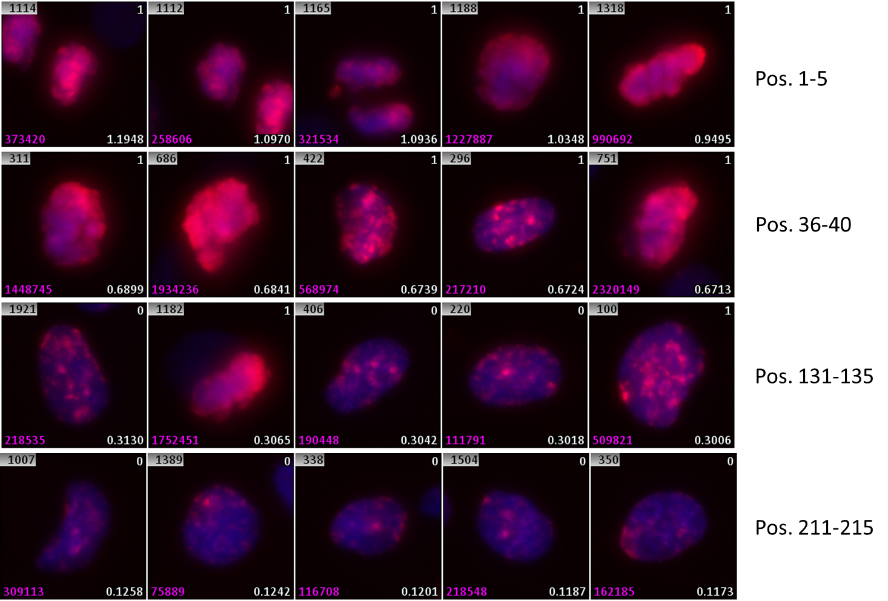


**Fig. S7:** **Representative pH3 (S10) stained WS1 cell nuclei, sorted by the area parameter.** Since the feature is normalized to the size of the nuclei, it is not surprising that this sorting is led by Anna and Telophases.


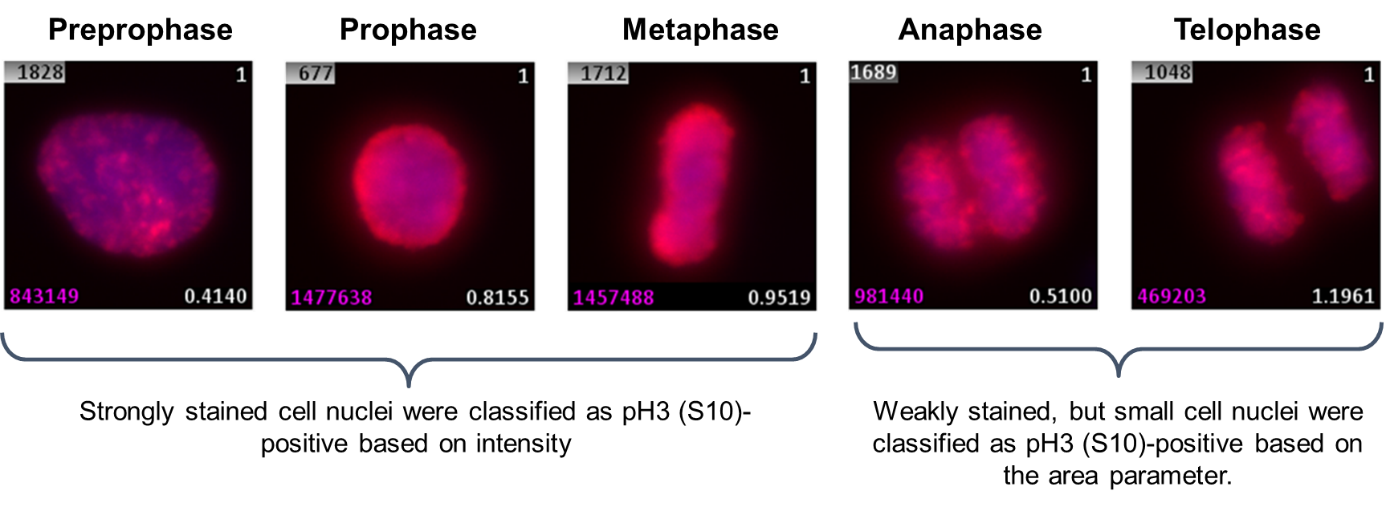


**Fig. S8:** **pH3 (S10) staining during progressing mitosis of WS1 cells.** pH3 (S10) is given in red, with a DAPI counterstained in blue. The cell number is shown at the top left, the intensity of the pH3 (S10) staining at the bottom left, the value of the area parameter at the bottom right, and the classification (1 = positive) is shown at the top right.


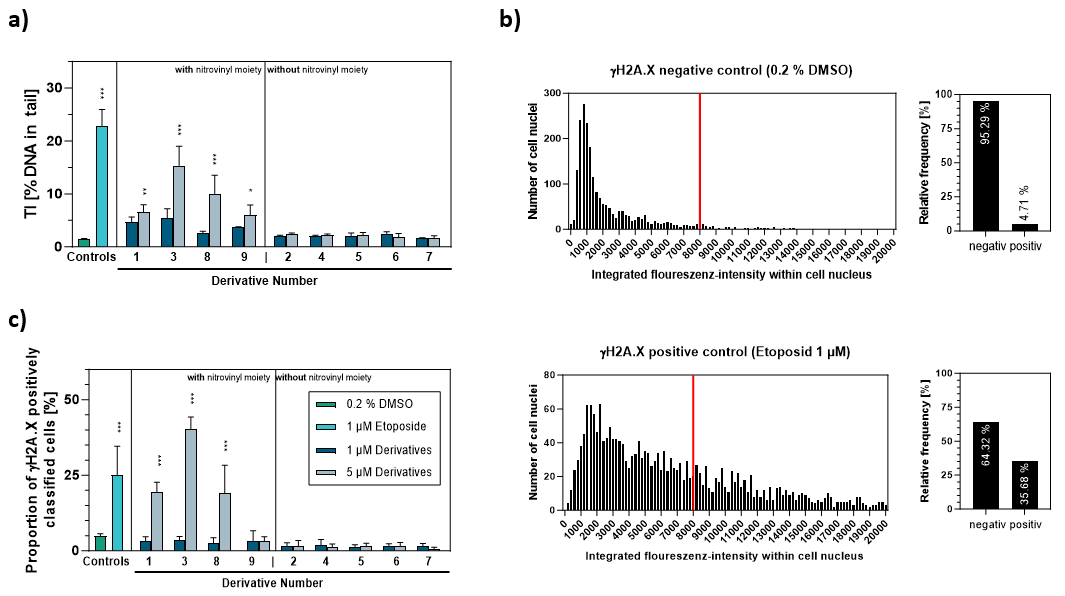


**Fig. S9**: Representative results of the γH2A.x classifier for DMSO and Etoposide treated WS1 cells. On the left, the integrated fluorescence intensity is given. Nuclei were quantified positively if the fluorescence intensity was above the threshold of 8000 units, depicted as a red line. The resulting proportions of classified nuclei are shown on the left.

Based on a threshold (red line in Fig. S9) of 8000 Florescence Units, cell nuclei were classified positively (staining intensity is above the threshold) or negatively (staining intensity is below the threshold) and as displayed in Fig. S9.


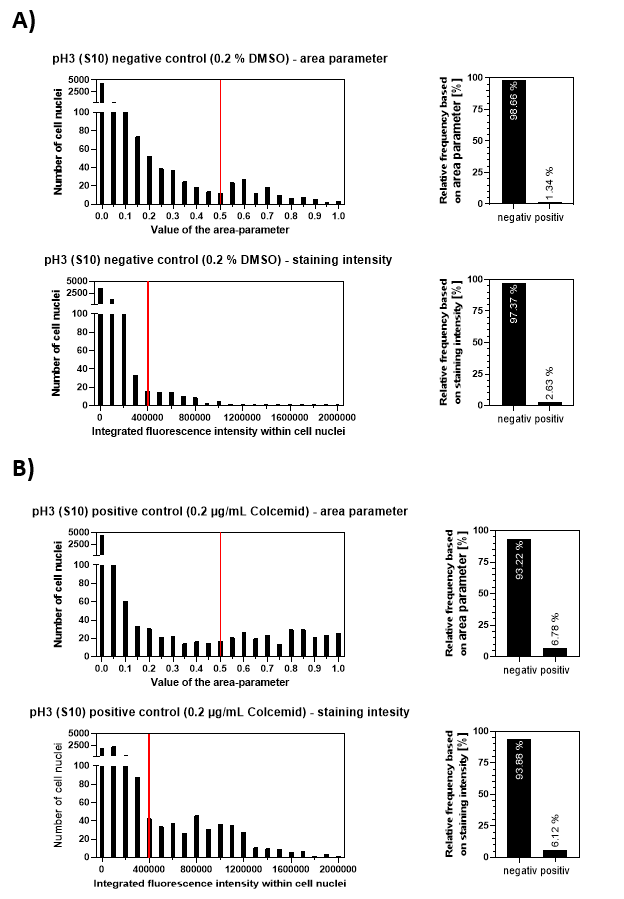


**Fig. S10: pH3 (S10)** Representative results of the pH3 (S10) classifier for DMSO and Colcemid treated WS1 cells Classifier details of **A)** vehicle and **B)** positive control. The classification is based on two aspects, first being the fluorescence intensity, shown in the upper part of **A)** and **B)** and the second being an area parameter, displayed in lower half. Nuclei quantified above the threshold of 0.5 for the area parameter or 400.000 for the fluorescence intensity, respectively, were classified positively.

The pH3 (S10) classifier consists of two parts, which are show in Fig. S10. The first one, similar the γH2A.X classifier, assess the staining intensity of pH3 (S10). Nuclei above the set threshold of 400.000 fluorescence units were classified positively. The integrated pH3 (S10) fluorescence intensity of the positive and negative control is depicted in Fig. S10A and Fig. S10B in the upper section, respectively. As the staining intensity is decreasing with progressing mitosis as seen in Fig. S8 the staining intensity classifier was complemented by an area parameter, depicted in Fig. S10A and Fig. S10B in the lower section. For the area parameter, the threshold was set to 0.5.

The two folded classifier was necessary to positively classify cells during the ana- and telophase, as the staining intensity of Histone 3 fades with ongoing mitosis. Accounting for the reduction in nuclei area, the staining intensity was normalization for this area resulting in the area-parameter, highlighting the ana- and telophase (compare Fig. S6 to Fig. S8).


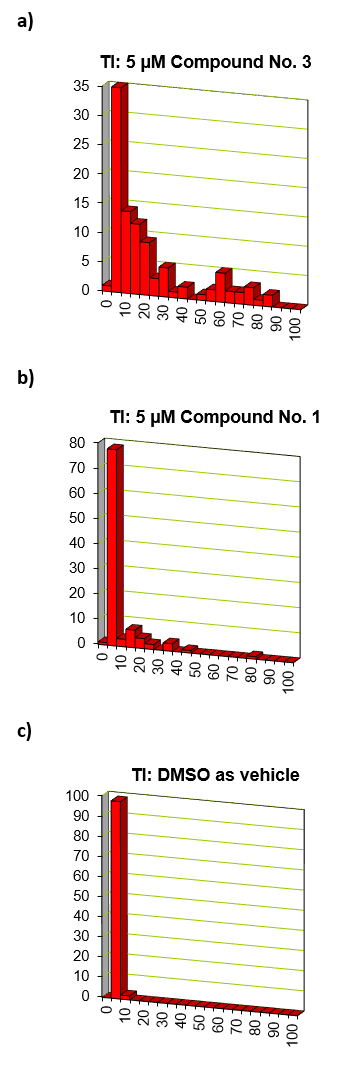


**Fig. S11:** Representative histograms of tail intensity distributions obtained during the alkaline comet assay. a) Compound no. 3 induced marked DNA-damages resulting in a bimodality distribution with the first peak ranging from 0 to 40 TI and the second from 50 to 90 TI. b) For compound no. 1 the first peak ranged from 0 to 10 TI with an additional peak from 10 to 30 TI. c) The bimodality was not present in the vehicle (DMSO) control.
